# Exploring the experiences of the hemodialysis patients during the COVID-19 pandemic: A qualitative study

**DOI:** 10.12688/f1000research.158482.1

**Published:** 2024-11-21

**Authors:** Dewiyanti Toding, Masfuri Masfuri, Agung Waluyo, Sri Yona, Reflin Mahmud, Sri Nining, Effita Piscesiana, Wasal Desrial Siregar, Rico Maulana Nugroho, Ropika Ningsih, Ida Ayu Md Vera Susiladewi

**Affiliations:** 1 Dialysis Unit, Hasanuddin University Hospital, Makassar, South Sulawesi, 90245, Indonesia; 2 Faculty of Nursing, Universitas Indonesia, Depok, West Java, 16424, Indonesia; 3 Nursing Committee, Woman and Child Bunda Jakarta, Menteng, Central Jakarta, 10350, Indonesia; 4 Faculty of Nursing, Universitas Kristen Krida Wacana, West Jakarta, Jakarta, 12120, Indonesia; 5 Catheterization Laboratory, Universitas Airlangga Hospital, Surabaya, East Java, 60115, Indonesia; 6 Cardiovascular Center, Soeradji Tirtonegoro Hospital, Klaten, Central Java, 57424, Indonesia; 7 Nursing Study Program, Muhammadiyah University West Sumatra, Bukittinggi, West Sumatra, 26131, Indonesia

**Keywords:** COVID-19, end-stage renal disease, hemodialysis, pandemic, patient experience, nurse, qualitative descriptive

## Abstract

**Background:**

The impact and changes due to the COVID-19 pandemic on end-stage renal disease patients undergoing hemodialysis affect the patient’s fulfilment and quality of life.

**Objective:**

This study aims to thoroughly investigate the experiences of end-stage renal disease patients undergoing hemodialysis during the COVID-19 pandemic.

**Methods:**

This research uses a qualitative descriptive approach with in-depth interview methods. Participants numbered 15 people from Wahidin Sudirohusodo Hospital and Hasanuddin University Hospital, which were selected using a purposive sampling technique.

**Result:**

There are three themes produced in this research: 1) various responses to the COVID-19 pandemic, 2) various impacts, and 3) coping mechanisms developed.

**Conclusion:**

These findings indicate that patients with end-stage renal disease who undergo hemodialysis have attempted to build adaptive coping strategies in this era of the COVID-19 pandemic. However, they still need support from healthcare providers in the hemodialysis unit to overcome various problems and impacts resulting from the COVID-19 pandemic. Hemodialysis nurses are expected to be able to carry out holistic assessments and continuous evaluation in order to provide comprehensive nursing care to meet patient needs for hemodialysis in the era of the COVID-19 pandemic.

## Introduction

Patients with end-stage renal disease must undergo renal replacement therapy to continue life, and hemodialysis is one of the most common renal replacement therapies carried out throughout the world, including in Indonesia ( Liew, 2018). End-stage renal disease patients are classified as a high-risk group for being infected with COVID-19 because they have several risk factors, such as advanced age, decreased immune status, diabetes, heart disease, and hypertension ( Onder et al., 2020; Xiong et al., 2020; Valeri et al., 2020). Hemodialysis therapy is generally carried out in central hemodialysis units where patients, nurses, doctors and support staff can be present simultaneously during the hemodialysis process, so it is sometimes difficult to maintain social distance and restrictions ( Rombolà & Brunini, 2020). Therefore, hemodialysis units have a high risk of COVID-19 transmission, including staff, fellow patients and the patient’s family ( La Milia et al., 2020; Su et al., 2020). Li et al. (2020) said that fear and anxiety about being exposed to COVID-19 are also a severe challenge that hemodialysis patients must face. As a risk group, hemodialysis patients have a fear of not being able to face COVID-19 if they are exposed. As a high-risk group, hemodialysis patients fear they may not be able to cope with COVID-19 if exposed ( WHO, 2020a). This concern is heightened by the limited availability of personal protective equipment in hospitals or for personal use, which can further increase their levels of anxiety and fear. Hemodialysis patients can experience changes in behaviour towards medication adherence during the COVID-19 pandemic ( Sousa et al., 2020).

Hemodialysis patients should also take extra precautions to minimize the risk of exposure to the COVID-19 virus ( Henry & Lippi, 2020). Hemodialysis patients must go to the unit hemodialysis 2-3 times per week and undergo 4-5 hour hemodialysis process, further increasing the patient’s risk of being exposed to COVID-19 ( Su et al., 2020). Transportation to the hospital is also a challenge, as many hemodialysis patients have to face patients who depend on public transportation ( Verma et al., 2020). Meijers, Messa, and Ronco (2020) suggest that hemodialysis patients use private transportation to the hemodialysis unit to reduce the contact risk of exposure to COVID-19. The COVID-19 pandemic can affect end-stage renal disease patients undergoing the process of hemodialysis and its treatment therapy. What is the condition of hemodialysis patients in Indonesia? During the COVID-19 pandemic, not much has been studied, especially regarding patients experiencing hemodialysis during the COVID-19 pandemic. Understanding the hemodialysis process during the COVID-19 pandemic from the experience and perspective of hemodialysis patients will make it easier for nurses to understand the patients needs. Data and information about the experience of hemodialysis patients are essential for nurses when providing hemodialysis treatment during this COVID-19 pandemic, so the nursing care provided is more comprehensive.

## Methods

### Study design

This research used qualitative descriptive design that describes the experiences of end-stage renal disease patients undergoing hemodialysis in Indonesia in the era of the COVID-19 pandemic. This method is commonly used in health research and exploring various phenomena related to patient experiences ( Doyle, McCabe, Keogh, Brady, & McCann, 2020). A qualitative descriptive research design was selected based on the research objectives, which aim to describe the experiences of patients undergoing hemodialysis for end-stage renal disease during the COVID-19 pandemic in a direct, extensive, and information-rich manner. Each patient has a unique perspective on their hemodialysis experience during the pandemic. Therefore, the researchers aimsto capture these perspectives to develop more suitable nursing interventions for them.. Thus, a qualitative descriptive research design is well-suited for this study.

### Setting and participants

This research was conducted in February 22 to May 22 2021 at Hasanuddin University Hospital and Wahidin Sudirohusodo Hospital. Recruitment of participants in this research used the method of purposive sampling with inclusion criteria including (1) patients undergone hemodialysis for more than three months, (2) negative PCR swab test result (not confirmed COVID-19), (3) more than 18 years old, and (4) able to communicate in Bahasa. The exclusion criteria were hemodialysis patients with cognitive impairment. The determination of participants is based on data adequacy. Once data saturation is reached, whereby no new insights or information can be derived from the participants ( Bradshaw et al., 2017; Vasileiou et al., 2018).

### Data collection

Data collection method with in-depth interviews was used in this research. The interviewer was the first author, a dialysis nurse with 10 years of experience. She holds certification in dialysis nursing competency, master of nursing degree, has completed qualitative research courses and proficient in data collection through interviews. She has also practised conducting interviews with hemodialysis patients, though not with the specific participant.

The interview results were documented using voice record by phone to record participants’ verbal responses. The interview was conducted in person at the hospital and over the phone from home, in accordance with the prior agreement made with each patient before the interview.

Questions were focused on exploring the experiences of patients undergoing hemodialysis during the COVID-19 pandemic, following prepared guidelines for in-depth interviews. We employed a guideline-based interview approach, conducting semi-structured interviews with open-ended questions. In order to assess the efficacy of the interviewer and the interview protocol, we conducted preliminary interviews with three patients who were not part of the study. The outcomes of these preliminary interviews were documented and reviewed with the supervisors. The research proceeded only after obtaining supervisor approval to continue the interviews. Each interview was conducted individually, lasting between 60-90 minutes, with participants given the flexibility to take breaks or reschedule follow-up interviews as needed. Participants were interviewed individually to provide them with greater freedom to express themselves. When conducting interviews, the researcher remained attentive to research ethical principles and implementing health protocols. The interview ended on condition when the interview contract time had expired, the purpose of the interview had been achieved and the participants answers had become repetitive. Data saturation was achieved with the 15th participant, as no new information emerged, and therefore, no additional participants were recruited. No interviews were repeated in this study.

### Data analysis

Data analysis carried out in this research used inductive thematic analysis consisting of 6 stages, namely recognizing and transcribing data, coding data, compiling themes, reviewing themes that have been prepared, giving names and definitions to themes, and making reports on research results ( Braun et al., 2019). Every interview session was recorded and transcribed, and we used field notes to record participants’ expressions during the interview. Transcribed data were returned to participants for confirmation and clarification before being processed further. Additionally, the researchers’ considerations were documented throughout the analysis process to ensure the accuracy and validity of the data interpretation. NVivo 12 Plus was utilized for the analysis ( QSR International Pty Ltd. Nvivo, 2024). The findings were shared with participants for verification. Two experts oversaw the research, and its validity was assessed through a thesis examination involving two expert examiners and nursing master’s students as reviewers. This comprehensive process ensured the research’s quality and reliability.

## Result

The participants in this research were 15 people, consisting of 9 males and 6 females. No one asked for withdrawing. Participants in this study were aged 23-66 years and had varying education levels, namely elementary school, middle school, high school, diploma, and bachelor’s degree. Participants’ jobs varied from entrepreneurs, freelancers, teachers, farmers, private employees, and students, and 6 participants did not work. Participants in this study came from 3 large tribes in South Sulawesi, with 7 participants from the Bugis tribe, 6 participants from the Makassar tribe and 2 participants from the Toraja tribe. The participants had undergone hemodialysis therapy for a period of between 2-8 years, with the majority undergoing the therapy two times per week.

Participants have carried out swab examinations PCR 3-20 times. Researchers conducted face-to-face interviews three times and telephone due to the COVID-19 pandemic situation (can be seen in Table 1).

**Table 1.** Participant characteristics.

Participants	Length of time undergoing hemodialysis (years)	Schedule undergoing hemodialysis/week	Frequency of PCR swabs	Number of meetings
1	5	2 times	14 times	3 times
2	6	2 times	15 times	3 times
3	3	2 times	15 times	3 times
4	2	2 times	6 times	3 times
5	2	2 times	10 times	3 times
6	4	2 times	10 times	3 times
7	7	2 times	9 times	3 times
8	3	2 times	11 times	3 times
9	2	2 times	11 times	3 times
10	4	2 times	6 times	3 times
11	8	2 times	9 times	3 times
12	3	2 times	10 times	3 times
13	4	2 times	20 times	3 times
14	5	2 times	3 times	3 times
15	2	2 times	9 times	3 times

### Thematic analysis results

The experiences of end-stage renal disease patients undergoing hemodialysis in the era of the COVID-19 pandemic have yielded three overarching themes. These themes encompass the following: (1) the emergence of diverse responses at the onset of the pandemic, (2) the emergence of various impacts throughout the pandemic, and (3) the development of coping strategies during the pandemic.

***Theme 1: The emergence of diverse responses at the onset of the pandemic***

The research team identified the initial theme through an analysis of participant responses regarding their experiences with hemodialysis during the early stages of the pandemic. The findings revealed a range of negative reactions, which were grouped under the sub-theme “emotional response and behaviour response.”

*Emotional response*

At the outset of the pandemic, participants described a range of negative emotional responses. At the outset of the COVID-19 pandemic, individuals undergoing hemodialysis expressed concerns, apprehension, and distrust. Anxiety was reported by nearly all participants at the onset of the pandemic. The majority of participants expressed concern that they might encounter difficulties in accessing hemodialysis in the event of a positive test result for SARS-CoV-2. Additionally, the majority of participants expressed concern about developing symptoms similar to those observed in patients with COVID-19 at the onset of the pandemic. It is not uncommon for patients with chronic kidney failure undergoing hemodialysis to present with symptoms such as cough, shortness of breath, flu-like symptoms, and fever. Some participants indicated that they experienced feelings of anxiety in relation to both fellow hemodialysis patients and hemodialysis nurses. Such feelings of anxiety are a consequence of the suspicion that either the patient or the nurse may be carrying the SARS-CoV-2 virus.

*“This swab keeps making me scared because this also keeps my blood pressure rising” (P2).*

*“I am afraid because it will be difficult for me later. Finding hemodialysis schedules and entering isolation are problematic. It is hard if we get COVID-19” (P3).*

*“Had I been provided with the swab, I would have experienced a sense of unease and anxiety tend to ruminate on issues, which can precipitate a state of physical illness” (P4)*

*“We have a disease and are prone to be affected. That is why there is a disease that we carry. We were nervous about receiving the swab results” (P5).*

*“Frequently, I experienced feelings of anger and frustration. However, I refrained from expressing my emotions towards other patients. At most, I informed the nurse to request a swab test, as I was concerned about the potential risk of contracting a disease in this environment” (P13).*

*“I feared that I would not be provided with a hemodialysis machine, given the limited availability of such devices for use in patients with COVID-19” (P14).*

Furthermore, some participants articulated apprehension regarding the potential for testing positive for COVID-19 infection due to their classification as a high-risk group. This concern arises because patients with chronic kidney disease have an immune system that is compromised, rendering them more susceptible to infection by COVID-19, as expressed by the participants:

*“We are at higher risk because we have a pre-existing condition. That is why we are anxious” (P5).*

*“The result of the swab should not be positive because my fear is overwhelming. It is not just that we (hemodialysis patients) have kidney disease, but the doctors have said we are more susceptible, more at risk of contracting COVID-19” (P6).*

*“What worries me is the isolation room because no visitors are allowed. I feel bad because I cannot walk or do anything on my own, so I am worried about how I will manage inside if no one can visit” (P9).*

*Behavioural response*

In the initial stages of the global pandemic caused by COVID-19, individuals exhibited a range of behavioural responses to concerns, fears, and suspicions. Such behaviours include maintaining compliance with hemodialysis, concealing one’s health status, and disregarding social etiquette. This is exemplified by patients with dependent terminal chronic renal failure who undergo hemodialysis therapy to maintain survival. From the outset of the pandemic, participants expressed concern and apprehension about COVID-19. Nevertheless, all participants endeavoured to persevere with their hemodialysis regimen.

*“I’m concerned because my throat is itchy and I’ve been told that coughing is a symptom of corona. If I can treat it myself, I’d prefer to do so and not have to tell anyone else” (P2)*

*“what else can I do? This is my fate; I have to go to dialysis if I want to survive” (P15)*

Participants chose to hide their own or their family’s health status due to fear of being stigmatized as having COVID-19 or being in contact with someone who had it. For example, one participant mentioned:

*“I hope it’s just a regular cough because of an itchy throat and not labelled as COVID-19. If it’s something we can treat, let’s not jump to conclusions” (P2)*

*“I hid my husband from the nurse because I was afraid that my husband would be suspected, even though I was the one who was sick, my husband was not” (P3)*

Another behavioural response observed was a disregard for social norms and etiquette. Some participants exhibited a tendency to disengage from social interactions because they were excessively afraid of contracting COVID-19.

*“Friends who were close now keep their distance, saying ’stay away.’ They weren’t snobbish before, but now they are. I would rather that than catch COVID-19” (P2).*

*“I am careful about who comes into my house. I would rather offend someone than risk it. Neighbours who used to visit now stay away because I get angry at them. Let them be offended; I would rather be safe” (P3).*

***Theme 2: Various impacts***

The COVID-19 pandemic has had many impacts on various sectors of participants’ lives as patients with end-stage renal disease undergoing hemodialysis. These various impacts are summarized in the sub-themes of physical, psychological, social, and economic impacts and protocol implementation health experienced by participants during the COVID-19 pandemic.

*Physical impact*

The majority of participants reported experiencing physical effects, including weight gain during interdialytic periods, shortness of breath, elevated blood pressure, pain, discomfort, and bleeding. The most frequently reported issue was interdialytic weight gain. As a consequence of the pandemic, the patient’s hemodialysis schedule was postponed. It should be noted that swab results were not available. In addition, some patients’ schedules have been modified as a result of the hemodialysis unit’s efforts to optimize the hemodialysis schedule. Shortness of breath represents a physical impact experienced by participants as a consequence of delays or alterations to their hemodialysis schedules. An increase in blood pressure is also a physical impact experienced by hemodialysis patients during COVID-19 pandemic due to anxiety about swab results. Some participants exhibited considerable anxiety regarding the results. The swab yielded a positive result, which was accompanied by an increase in blood pressure. The subsequent physical impact is pain. The mandatory swab obligations carried out by participants are a cause of pain, which is necessary for them to continue undergoing the hemodialysis process.

*“My body feels heavy. They usually remove three litres of fluid, but now they only remove two or two and a half litres during the four-hour sessions” (P1).*

*“My stomach is bloated, and my legs are swollen. They removed three litres before, but now it is only two” (P2).*

Another physical impact reported by the participants was difficulty breathing, which was often the result of postponed or rescheduled hemodialysis sessions.

*“It gets really bad; I feel breathless, and it gets worse if I miss a session of hemodialysis” (P9).*

*“Yes, I feel breathless, especially if I have not had hemodialysis for a while” (P12).*

The subsequent physical impact observed was an increase in blood pressure due to anxiety over swab test results. Several participants experienced significant anxiety at the prospect of testing positive for COVID-19, which in turn led to elevated blood pressure. The following are quotes from some participants:

*“The swab test is what scares me, and it causes my blood pressure to rise. It is always on my mind, especially when waiting for the results—my results are coming out tomorrow, and I am so anxious” (P2).*

*“After a swab, I feel uneasy. Before the pandemic, my blood pressure was normal. Now it often spikes. It is still high and rarely goes down” (P4).*

The other physical impact is pain. This is a direct consequence of the mandatory swab, which participants must carry out in order to continue undergoing the hemodialysis process. The process of taking samples for swab examination, which occurs every 2-3 weeks, causes pain for some participants. The following statements from several participants illustrate this point.

*“The swab every 14 days hurts my nose” (P2).*

*“This swab is really painful. It is only a matter of time before I start screaming” (P9).*

*Psychological impact*

The COVID-19 pandemic has also had a significant psychological impact on patients undergoing hemodialysis. The necessity of awaiting the results of the swab and the obligation to undergo continuous swabs has resulted in the patient experiencing disturbances to their sleep patterns, a sense of being a burden, and a perception of being under constant observation during the course of this pandemic. The majority of participants reported experiencing disturbances in their sleep patterns during the pandemic. However, during the current pandemic, these disturbances have been exacerbated due to concerns about the results of the swab. The majority of participants reported feeling burdened by the obligation to undergo swab tests. The obligation to undergo swabbing every 2-3 weeks necessitates that participants visit a hospital outside of their scheduled hemodialysis appointment and other scheduled examinations. The process of swabbing is quite intricate and time-consuming. The extended period is a significant burden for participants, particularly given their history of complications related to declining kidney function. One participant also reported a psychological impact resulting from the constant observation by nurses.

*“When it is close to the following swab schedule, I often have trouble sleeping. The stress and anxiety that come with the condition make it difficult for me to get the rest I need. As my stress levels rise, I find it even harder to sleep” (P8).*

*“I’m so scared, I’m really worried. I hardly ever sleep at night. I’m always thinking about when the phone will ring, telling me I’m positive COVID-19” (P11).*

Another psychological impact was the sense of being overwhelmed by the frequent swab tests. The necessity of attending the hospital outside of their regular hemodialysis schedule introduced an additional source of stress to an already challenging situation. The process of scheduling and undergoing the tests was found to be overwhelming by the participants, as evidenced by the following description:

*“It’s such a burden to have to do the swab every 21 days. The whole process—the route, the queue, registering, then seeing the doctor the next day, and so on—it’s exhausting” (P12).*

Another participant echoed this statement:

*“The process of undergoing a swab is quite exhausting. It involves waiting in line, expressing dissatisfaction, sitting down, and then being called again, going to the emergency room, and finally, to the swab room. This cycle occurs every two weeks, and the fatigue associated with it persists” (P13).*

*“It feels like I’m always being watched during this pandemic. I keep wondering when I’ll have to swab again” (P9).*

*Social impact*

Additionally, several participants reported experiencing social impacts as a result of the pandemic. A reduction in social interaction and a sense of ostracism were among the social impacts experienced by participants. A reduction in social interaction has been observed among fellow hemodialysis patients in the hemodialysis unit. Other social impacts include instances of ostracism. Some participants were ostracised by their families and neighbours during the pandemic due to the assumption that they had contracted the COVID-19 virus from the hospital.

*“Upon arrival at the hemodialysis unit, I found it necessary to limit my interactions with others. I maintained a distance and focused on personal hygiene, such as cleaning my bed. The atmosphere was noticeably different from what I had experienced previously” (P2).*

The pandemic also had a profound social impact on participants, particularly in terms of social isolation.

*“We feel like we’re at a distance from our friends. Before, we could forget about our illness by spending time with friends, but now we have to keep our distance” (P7).*

Others were shunning another social impact. Some participants reported being avoided by their neighbours and family members due to fears that they might bring COVID-19 home from the hospital.

*“It’s frustrating when I visit neighbours, and they immediately leave when I arrive. I tell them I don’t have the virus, but they don’t listen. I have a swab test result every two weeks, but they ignore me” (P5).*

*“People think the hospital is full of COVID-19, so when I come home from the hospital, they avoid me” (P7).*

*Economic impact*

Additionally, several participants reported experiencing the economic consequences of the global pandemic caused by COVID-19 pandemic. The cost of living has risen for those who require hemodialysis therapy. The obligation to undergo swabbing procedures has resulted in an increase in the number of visits to the hospital by participants, necessitating the expenditure of funds on transportation. Furthermore, participants are required to bear additional costs associated with the procurement of personal protective equipment, including masks.

*“The issue is that wearing a mask is quite difficult. I believe masks are not meant to be used twice, so we have to replace them. If we’re on a tight budget, we have to use them twice. We used the first mask 2-3 times. It is much work. That’s why my husband is back in his hometown to earn money for hospital expenses” (P7).*

***Theme 3: Coping mechanisms developed***

*Adaptive coping*

Adaptive coping is the most prevalent coping strategy individuals employ in response to challenges encountered during the ongoing pandemic. Other coping adaptations employed by participants include spiritual strengthening.

*“I pray to God every day and night. Hopefully, we won’t get infected with COVID-19” (P11).*

*“We are sick, and the swabs make us feel worse, so we keep praying that these swabs will stop soon" (7).*

Some participants attempted to maintain a positive outlook despite the negative emotions they experienced during the pandemic.

*“I worry sometimes, but I know my immunity is good, so I just stay positive” (P4).*

*“I try to stay calm and face everything head-on. This is not something that comes easily to everyone, so I tell my friends that maybe we’re being tested. Alhamdulillah, it gives me strength, so I stay motivated” (P5).*

Another adaptive coping strategy was dietary change. Some participants modified their diets to forestall any complications before undergoing swab tests.

*“I’ve been trying to cut back on my fluid intake and improve my diet. I make sure to eat well every time I have a swab coming up” (P5).*

*“I changed my diet because I used to eat oily and fried foods. Since the pandemic, I avoid them. I don’t eat ice cream anymore. I’m afraid of getting short of breath before the swab, so I limit my diet” (P7).*

Furthermore, participants demonstrated an ability to adapt to the revised schedule and the necessity of utilizing personal protective equipment (PPE) during hemodialysis.

*“When I first started, I was always late for my hemodialysis schedule in the morning, but after a couple of weeks, I got into a routine and stopped being late in the morning” (P1).*

*“I have become accustomed to the new protocol. Initially, I found it unusual to be instructed to wear a mask, but I have since made it a routine practice. It is now unusual for me not to wear a mask at the hospital” (P14).*

*Maladaptive coping*

While some participants employed adaptive coping strategies in response to the various challenges and difficulties encountered during the ongoing pandemic, others resorted to maladaptive coping mechanisms to deal with the pandemic’s multifaceted impacts. Some participants employed excessive prevention and withdrawal mechanisms as a means of adapting to the conditions of the pandemic.

*“I drank vitamin C, folic acid and played sports despite the rain. I drink honey and boiled ginger, which are high in potassium, but I do not care. The main thing is that it is clear: do not be exposed to COVID-19” (P3).*

*“I slept with a mask on. My child asked why I wore a mask to sleep. I said it was because I was afraid of COVID-19. I also bought vitamins from the pharmacy because I was scared.” (P6).*

Another maladaptive coping strategy observed was social withdrawal during hemodialysis. Some participants elected to withdraw from social interactions in order to circumvent the stigma associated with their condition. As individuals with end-stage renal disease, they were required to undergo hemodialysis two to three times per week at the hospital. However, the stigma associated with COVID-19 prompted them to avoid contact with others.

*“I do not want to be identified as a patient with COVID-19. When I am in the hospital, I do not talk to or touch other people” (P4).*

*“I’d rather stay silent than explain myself. If people misunderstand, I accept it. I can’t change what they think” (P10).*

## Discussion

Stress during an infectious disease outbreak include fear and worry about health problems and worsening chronic health problems ( CDC, 2020). Entire participants in this study also expressed various negative emotions, such as feelings of worry, fear, and suspicion, in line with research conducted by Xia et al. (2020), which shows the high level of psychological distress experienced by hemodialysis patients at the start of the pandemic, this was due to the high frequency of patients going to the hospital to undergo hemodialysis therapy and concerns about the transportation used. Other research that supports this argument conducted by Guerraoui et al. (2021), which shows the occurrence of anxiety and depression in hemodialysis patients in the early days of the pandemic due to fear of getting infected with COVID-19 due to the high incidence of COVID-19 and fear of transmission of COVID-19 to their family.

At the start of the pandemic, most participants were worried about COVID-19, causing participants to have difficulties accessing hemodialysis center. Hemodialysis schedule were overcrowding and limited health personnel in hemodialysis units which made this situation more challenging to prepare many special machines for hemodialysis patients with COVID-19 at the start of this pandemic ( IRR, 2018). Some participants also felt worried about being ostracized if infected with COVID-19. Infectious diseases are usually associated with stigma and cause discrimination against this group of patients in society ( Abdelhafiz & Alorabi, 2020). Pandemics like COVID-19 cause fear and anxiety, which can lead to social stigma that can ultimately lead to the occurrence of social avoidance ( Turner-Musa et al., 2020). Common symptoms of COVID-19, such as fever, cough, and shortness of breath, were common symptoms that hemodialysis patients experienced before the pandemic ( IRR, 2018). There are similarities in symptoms between COVID-19 and symptoms commonly experienced by hemodialysis patients, which also ultimately caused the emergence of suspicion among hemodialysis patients towards other hemodialysis patients.

Previous research shows that patients experienced feelings of worry and fear that hemodialysis can make them non-compliant with medication therapy, including non-compliance in undergoing hemodialysis therapy ( Kimmel, 2001; Kimmel & Peterson, 2005). However, this is different from what was obtained in this study. Amid worry, fear, and suspicion at the start of the pandemic, most hemodialysis patients still stated they were trying to maintain their compliance in undergoing hemodialysis therapy. Participants stated that the impact of not undergoing hemodialysis is more significant than not undergoing hemodialysis due to their worries and fears about COVID-19. They depend on hemodialysis therapy to maintain continuity in their lives and prevent the emergence of various further complications due to their disease condition if they do not comply with medical therapy.

Excessive emotional responses can cause negative behavior. COVID-19 is a new infectious disease that can cause anxiety and fear excessively, which can lead to dangerous behaviour ( Ho et al., 2020). This study found that worry, fear, and suspicion experienced by participants in the early days of the COVID-19 pandemic led to the emergence of several negative behaviors from participants, such as hiding their health status and ignoring social ethics. Participants chose to hide their health status, mainly if they got symptoms that were similar to the main symptoms of COVID-19 or had a history of contact with families who have COVID-19 symptoms. They done it to avoid referrals to an isolation room and carrying out COVID-19 screening which could later have an impact on delay in the participant’s hemodialysis schedule. Therefore, if participants already have symptoms such as fever, cough, and shortness of breath, participants will chose to treat themselves. This argument is supported by research conducted by Villa et al. (2020), who stated that stigma can lead people from possibly suffering from COVID-19, hiding symptoms to avoid pressure from the surrounding environment. This can make patients who have mild symptoms to avoid treatment from the health unit and act as usual so as not to arouse suspicion about their condition. When a crisis or pandemic occurs, individual or community will look for information about what happened. However, if information from trusted sources or official news is limited, people will look for information on social media and other media, which the truth cannot be guaranteed . This can cause a negative emotional response in a society that tends to experience extreme fear and uncertainty and negative behaviour is often driven by fear and distorted risk perceptions ( Torales et al., 2020). Research conducted by Wang et al. (2020a) stated that the dissemination of health information related to COVID-19, continuously updated and verified for accuracy by authorities or trusted parties, can reduce levels of anxiety, stress, and depressive symptoms in society. Therefore, the role of the nurse as an educator is vital.

The COVID-19 pandemic has impacted physical health, mental health, social life, and the economy. Patients with chronic diseases will experience many impacts, especially health problems during the COVID-19 pandemic caused by delays or difficulties in scheduling health services ( Singh et al., 2021). WHO (2020) revealed that more than half of the countries affected by COVID-19 reported many disruptions to care services for patients with the disease. Chronic, especially those requiring routine or long-term treatment. It was found in this research that the COVID-19 pandemic had a physical impact on kidney failure patients undergoing hemodialysis due to delays and schedule changes in hemodialysis. The COVID-19 pandemic not only causes many physical health impacts, but it also has many mental health impacts ( Pfefferbaum & North, 2020).

The mental health and psychosocial impacts of the COVID-19 pandemic may be more severe among people with chronic diseases. In the presence of chronic diseases, they are a group that can have severe complications if they infected by COVID-19, causing increased perceived stress and ultimately making worse their health problem ( WHO, 2020). Participants also experienced various psychological impacts during the pandemic. High stress due to concerns about being positive for COVID-19 from the final swab examination results caused almost all participants to experience sleep disturbances, increased blood pressure, burden, and feeling shunned during this pandemic. The COVID-19 pandemic can have a long-term impact on the physical and mental health of hemodialysis patients. Currently, The World Health Organization (WHO) is actively trying to control and reduce the impact of this pandemic by identifying, testing, and treating infected patients and developing drugs, vaccines, and treatment protocols ( WHO, 2020b). However, success in overcoming the pandemic cannot be confirmed ( Kumar & Nayar, 2020). Chronic disease sufferers can face lifestyle disturbances due to the COVID-19 outbreak, physical activity, sleep, stress, and mental health, which need to be handled better ( Kendzerska et al., 2021). Research conducted by Yang et al. (2021) shows a relationship between mental status changes and hemodialysis patients’ quality of life during the COVID-19 pandemic. Therefore, it is necessary to provide psychotherapeutic interventions during this pandemic to improve the quality of life of hemodialysis patients.

Hemodialysis patients also experience social impacts. Most participants stated there is decreased social interaction between fellow hemodialysis patients. Research conducted by Yang et al. (2021) also stated that there was increased social dysfunction in patients hemodialysis since the beginning of the pandemic. Therefore, hemodialysis nurses can create chat groups that are innovative, engaging, and easily accessible to hemodialysis patients and families.

The COVID-19 pandemic increases the cost burden on hemodialysis patients, mainly by supplying funds for purchasing personal protective equipment. Apart from that, swabs and other examinations are mandatory. The pandemic caused an increase in the frequency of hospital visits, this has increased the amount of personal protective equipment that must be prepared and increased costs for transportation. Research conducted by Lee et al. (2020) also shows that 90% of patients in hemodialysis are concerned about the economic impact and difficulties of finances during the COVID-19 pandemic. Adjusting swab examination schedules and other examinations with hemodialysis schedules may be a dialysis unit consideration. Reducing the frequency of going to the hospital can reduce transportation costs. Hemodialysis patients can simultaneously reduce the risk of exposure to COVID-19 both during travel and in the hospital.

Participants in this research also developed various coping strategies to deal with the various stressors experienced in the COVID-19 pandemic. Most participants used spiritual strengthening, thinking positively, and trying to accept and get used to it as adaptive coping mechanisms during the pandemic. Several previous studies also show that there are efforts to implement adaptive coping strategies in facing the COVID-19 pandemic. It is like relying on increased social support ( Cao et al., 2020) and adopting preventive measures to offset or minimize health risks and finances brought about by COVID-19 ( Wang et al., 2020a). Maladaptive coping refers to coping strategies associated with poor mental health outcomes and symptoms of higher levels of psychopathology, such as avoidance and emotional suppression ( Compas et al., 2020). In this research, several participants also developed maladaptive coping during the pandemic, include taking excessive preventive actions and withdrawing hemodialysis to face various stressors during the pandemic. Several previous studies identified factors of demographics as a potential risk of implementing maladaptive coping strategies in dealing with the COVID-19 pandemic, such as age, gender, race, socioeconomic status, and the burden of being parents ( Atchison et al., 2021; Clay & Parker, 2020). However, this study did not find a relationship between demographic data and participants’ maladaptive coping strategies. Therefore, differences in the participants’ personalities can be a factor in using coping strategies in this research. This is relate to the research conducted by Bacon & Corr (2020) and Carvalho et al. (2020) state that personality functions influence adaptive and coping strategies related to health problems during the COVID-19 pandemic.

How individuals face and deal with situations caused by COVID-19 can impact their mental, physical health and their willingness to participate actively in much-needed action ( Brailovskaia & Margraf, 2020; Yang et al., 2020). The COVID-19 pandemic continues, and it is not known when it will end, including in this continuing era. According to Roy’s theory, during the COVID-19 pandemic, various stimuli, such as the various impacts experienced by hemodialysis patients, can influence the adjustment or adaptation of hemodialysis patients to their disease conditions. The research results show that most participants have attempted to adapt to various aspects of the impact of the COVID-19 pandemic, this can be seen from the participants’ efforts to maintain adherence to hemodialysis therapy amidst worry and fear against COVID-19. Most participants have also tried to build adaptive coping strategies in dealing with the various impacts of the COVID-19 pandemic. Therefore, hemodialysis nurses are expected to continue to support participants who strive to implement adaptive coping strategies and can help other participants experiencing maladaptive coping strategies to adaptive coping. Individuals with positive coping strategies usually show fewer symptoms of anxiety and stress ( Moccia et al., 2020; Wang et al., 2020b).

Additionally, hemodialysis nurses are expected to educate hemodialysis patients about appropriate coping skills that can significantly impact how they view their condition, the severity of symptoms, and psychological stress related to the COVID-19 pandemic. Understanding strategy coping is very important to support the patient’s coping efforts. Apart from that, it is necessary to monitor the patient’s coping strategies and evaluate the patient’s psychological status periodically so that the nursing care provided can be adjusted to current conditions and help hemodialysis patients to remain able to adapt in this pandemic situation so that their quality of life can still be maintained.

## Conclusion

Results of research on end-stage renal disease patients undergoing hemodialysis in the era of the COVID-19 pandemic illustrate three main themes, consist of (1) the emergence of various responses to the beginning of the pandemic both through emotional and behavioral responses, (2) the emergence of various impacts during a pandemic such as physical, psychological, social and economic impacts and (3) the existence of coping strategies that were built during the pandemic, namely adaptive and maladaptive coping. These findings showed that end-stage renal disease patients underwent hemodialysis in the COVID-19 pandemic era requires support from healthcare providers in hemodialysis units to overcome various problems and impacts resulting from the COVID-19 pandemic.

### Limitations

Due to the COVID-19 pandemic, interviews were limited to face-to-face sessions with 4 participants and phone interviews with 11 participants, based on their comfort levels. Health protocols during face-to-face interviews sometimes led to unclear responses, requiring follow-up questions, which may have affected the depth of the data. Additionally, the reliance on phone interviews limited the observation of non-verbal cues, potentially impacting the richness of the insights. The study’s findings are also based on a specific sample from two hospitals in Indonesia, which may limit their generalizability to other populations and contexts.

### Implications of this study

**Clinical practice implications**

Healthcare providers, especially hemodialysis nurses, should offer holistic care addressing both physical and psychological needs to reduce patients’ anxiety and stress.

**Health policy implications**

Health policies should prioritize preparedness for future pandemics by ensuring flexible care schedules and maintaining access to essential treatments, minimizing disruptions to patient care in times of crisis. Continuous evaluation of healthcare systems’ resilience is necessary to protect vulnerable patients in any future public health emergencies.

**Research implications**

This study is a valuable reference for developing bio-psycho-social interventions tailored to chronic kidney failure patients receiving hemodialysis, particularly during pandemic conditions. Further studies are needed to explore factors influencing patients’ coping strategies and to evaluate the long-term effects of the pandemic on the quality of life of dialysis patients.

## Ethical considerations 

Research ethics were ensured through the approval of the Faculty of Nursing Ethics Committee at the University of Indonesia, documented under ethical approval number: SK-15/UN2.F12.DI.2.1/ETIK 2021, dated February 3rd, 2021. The researchers provided explanations regarding the procedures, objectives, benefits, potential risks, and the rights and responsibilities of participants through a research information sheet. Participation was voluntary, as indicated by the signing of informed consent. Participants were also assured that their involvement was voluntary and were informed of their right to withdraw from the study at any time without facing any penalties.

## Data Availability

The interview transcripts cannot be publicly shared because the data contain sensitive information that could potentially identify the participants. The restriction is a guideline set by the ethics committee and included in the informed consent that participants have agreed to. Access to the data is restricted to protect participant confidentiality. Any data access requests must be reviewed and approved by the authors and will only be granted under conditions that ensure participant anonymity. All transcripts are in Bahasa. Those interested in reading the summary report, including quotes, may contact the corresponding author ( dewiyantitodinguh@gmail.com) for translations of the requested sections. Fighshare: COVID-19 pandemic: A Qualitative Study. figshare. Journal contribution, https://doi.org/10.6084/m9.figshare.27310995.v2 ( Toding, 2024). This project contains the following extended data:
a.
**Research explanation**: This section provides participants with details on the study’s objectives, methods, potential benefits, risks, and their rights. It also explains data usage and protection measures, along with how participants can ask questions or choose to withdraw from the study if necessary b.
**Informed consent**: this is the voluntary consent participants provide after being fully briefed on the study’s details, as outlined in the research explanation provided to them. c.
**Reporting guidelines**: we used COREQ checklist Data are available under the terms of the Creative Commons Attribution 4.0 International license (CC-BY 4.0).
