# Exploring the experiences of the hemodialysis patients during the COVID-19 pandemic: A qualitative study

**DOI:** 10.12688/f1000research.158482.2

**Published:** 2025-09-22

**Authors:** Dewiyanti Toding, Masfuri Masfuri, Agung Waluyo, Sri Yona, Reflin Mahmud, Sri Nining, Effita Piscesiana, Wasal Desrial Siregar, Rico Maulana Nugroho, Ropika Ningsih, Ida Ayu Md Vera Susiladewi

**Affiliations:** 1 Dialysis Unit, Hasanuddin University Hospital, Makassar, South Sulawesi, 90245, Indonesia; 2 Faculty of Nursing, Universitas Indonesia, Depok, West Java, 16424, Indonesia; 3 Faculty of Nursing, Universitas Kristen Krida Wacana, West Jakarta, Jakarta, 12120, Indonesia; 4 Nursing Committee, Woman and Child Bunda Jakarta, Menteng, Central Jakarta, 10350, Indonesia; 5 Catheterization Laboratory, Universitas Airlangga Hospital, Surabaya, East Java, 60115, Indonesia; 6 Cardiovascular Center, Soeradji Tirtonegoro Hospital, Klaten, Central Java, 57424, Indonesia; 7 Nursing Study Program, Muhammadiyah University West Sumatra, Bukittinggi, West Sumatra, 26131, Indonesia

**Keywords:** COVID-19, end-stage renal disease, hemodialysis, pandemic, patient experience, nurse, qualitative descriptive

## Abstract

**Background:**

Hemodialysis patients are highly vulnerable during the COVID-19 pandemic and face multiple challenges that affect their physical, psychological, social, and economic well-being.

**Objective:**

This study aims to thoroughly investigate the experiences of end-stage renal disease patients undergoing hemodialysis during the COVID-19 pandemic.

**Methods:**

A qualitative descriptive design was applied. Fifteen participants were recruited purposively from Wahidin Sudirohusodo Hospital and Hasanuddin University Hospital. Data were collected through in-depth interviews and analyzed thematically, guided by Roy’s Adaptation Model to capture adaptive and maladaptive responses.

**Result:**

There are three themes produced in this research: 1) various responses to the COVID-19 pandemic, 2) various impacts, and 3) coping mechanisms developed.

**Conclusion:**

The findings underscore the importance of holistic nursing interventions, psychosocial support, and continuous professional development for dialysis nurses to strengthen patient resilience during current and future health crises.

## Introduction

Patients with end-stage renal disease must undergo renal replacement therapy to continue life, and hemodialysis is one of the most common renal replacement therapies carried out throughout the world, including in Indonesia ( Liew, 2018). End-stage renal disease patients are classified as a high-risk group for being infected with COVID-19 because they have several risk factors, such as advanced age, decreased immune status, diabetes, heart disease, and hypertension ( Onder et al., 2020; Xiong et al., 2020; Valeri et al., 2020). Hemodialysis therapy is typically performed in central hemodialysis units, where patients, nurses, doctors, and support staff are all present during the procedure. As a result, it can be challenging to maintain social distancing and other restrictions ( Rombolà and Brunini, 2020). Therefore, hemodialysis units have a high risk of COVID-19 transmission, including staff, fellow patients and the patient’s family ( La Milia et al., 2020; Su et al., 2020). Li et al. (2020) said that fear and anxiety about being exposed to COVID-19 are also a severe challenge that hemodialysis patients must face. As a high-risk group, hemodialysis patients are concerned about their ability to cope with COVID-19 if exposed. This worry is intensified by the limited availability of personal protective equipment, both in hospitals and for personal use, which can further elevate their anxiety and fear. Furthermore, hemodialysis patients may experience changes in their behavior regarding medication adherence during the COVID-19 pandemic ( Sousa et al., 2020).

Hemodialysis patients should also take extra precautions to minimize the risk of exposure to the COVID-19 virus ( Henry & Lippi, 2020). They must go to the hospital 2-3 times per week and undergo 4-5 hour in hemodialysis process, further increasing the patient’s risk of being exposed to COVID-19 ( Su et al., 2020). Transportation to the hospital is also a challenge, as many hemodialysis patients have to face reality that they depend on public transportation ( Verma et al., 2020). Meijers, Messa, and Ronco (2020) suggest that hemodialysis patients use private transportation to the hemodialysis unit to reduce the contact risk of exposure to COVID-19. The COVID-19 pandemic can affect end-stage renal disease patients undergoing the process of hemodialysis and its treatment therapy. What is the condition of hemodialysis patients in Indonesia? During the COVID-19 pandemic, not much has been studied, especially regarding patients experiencing hemodialysis during the COVID-19 pandemic. Understanding the hemodialysis process during the COVID-19 pandemic from the experience and perspective of hemodialysis patients will make it easier for nurses to understand the patients needs. Data and information about the experience of hemodialysis patients are essential for nurses when providing hemodialysis treatment during this COVID-19 pandemic, so the nursing care provided is more comprehensive.

## Methods

### Study design

This research used qualitative descriptive design that describes the experiences of end-stage renal disease patients undergoing hemodialysis in Indonesia in the era of the COVID-19 pandemic. This method is commonly used in health research and exploring various phenomena related to patient experiences ( Doyle, McCabe, Keogh, Brady, & McCann, 2020). A qualitative descriptive research design was selected based on the research objectives, which aimed to describe the experiences of patients undergoing hemodialysis for end-stage renal disease during the COVID-19 pandemic in a direct, extensive, and information-rich manner. Each patient has a unique perspective on their hemodialysis experience during the pandemic. Therefore, the researchers aims to capture these perspectives to develop more suitable nursing interventions for them. Thus, a qualitative descriptive research design is well-suited for this study.

### Setting and participants

This research was conducted in February 22 to May 22 2021 at Hasanuddin University Hospital and Wahidin Sudirohusodo Hospital. Recruitment of participants in this research used the method of purposive sampling with inclusion criteria including (1) patients undergone hemodialysis for more than three months, (2) negative PCR swab test result (not confirmed COVID-19), (3) more than 18 years old, and (4) able to communicate in Bahasa. The exclusion criteria were hemodialysis patients with cognitive impairment. The determination of participants is based on data adequacy. Adequate data refers to data that is sufficient to address the research questions, indicated by the achievement of data saturation, where no new information or insights can be obtained from the participants ( Bradshaw et al., 2017; Vasileiou et al., 2018). Data saturation was determined independently at each location, based on the absence of new information that corresponded with the unique characteristics of the participants.

### Data collection

The data collection method used in this research is in-depth interviews (in-depth interviews). The interview results were documented using a tape recorder to record participants’ verbal responses. The interview was conducted in person at the hospital and via phone from home, following the prior agreement made with each patient before the interview.

Questions were focused on exploring the experiences of patients undergoing hemodialysis during the COVID-19 pandemic, following prepared guidelines for in-depth interviews. We employed a guideline-based interview approach, conducting semi-structured interviews with open-ended questions. In order to assess the efficacy of the interviewer and the interview protocol, we conducted preliminary interviews with three patients who were not part of the study. The outcomes of these preliminary interviews were documented and reviewed with the supervisors. The research proceeded only after obtaining supervisor approval to continue the interviews. Each interview was conducted individually, lasting between 60-90 minutes, with participants given the flexibility to take breaks or reschedule follow-up interviews as needed. Participants were interviewed individually to provide them with greater freedom to express themselves. When conducting interviews, the researcher remained attentive to research ethical principles and implementing health protocols. The interview concluded when the agreed-upon time had expired, the purpose of the interview had been met, and the participants showed signs of fatigue. Data saturation was reached with the 15th participant, as no new information emerged, and no additional participants were recruited. No interviews were repeated in this study.

Rigor of this research was using framework of Lincoln and Guba ( Gunbayi, 2024). Credibility implemented by conducted preliminary interviews with three patients who were not part of the study. Researcher spent 6 weeks in average in each site to gain thrustworthies with all participants. The interviewer was the first author, a dialysis nurse with 10 years of experience. She holds certification in dialysis nursing competency, master of nursing degree, has completed qualitative research courses and proficient in data collection through interviews. Two experts oversaw the research, and its validity was assessed through a thesis examination conducted by two expert examiners, with nursing master’s students serving as reviewers. This comprehensive process ensured the research’s quality and reliability. The research proceeded only after obtaining expertise approval to continue the interviews. Transcribed data were returned to participants for confirmation and clarification before being processed further. To ensure transferability in this research, we provided clear and detailed information about the content about the study. Additionally, all statements originally made in local languages have been translated into English to ensure that the research is accessible and understandable to all readers.

### Data analysis

Data analysis carried out in this research used inductive thematic analysis consisting of 6 stages, namely recognizing and transcribing data, coding data, compiling themes, reviewing themes that have been prepared, giving names and definitions to themes, and making reports on research results ( Braun et al., 2019). Every interview session was recorded and transcribed, and we used field notes to record participants’ expressions during the interview. Transcribed data were returned to participants for confirmation and clarification before being processed further. Additionally, the researchers’ considerations were documented throughout the analysis process to ensure the accuracy and validity of the data interpretation. NVivo 12 Plus was utilized for the analysis ( QSR International Pty Ltd. Nvivo, 2024). The findings were shared with participants for verification.

## Result

The participants in this research were 15 individuals, consisting of 9 males and 6 females. No one requested to withdraw from the study. The participants, aged 23-66 years, had varying levels of education, including elementary school, middle school, high school, diploma, and bachelor’s degree.

Their occupations varied, including entrepreneurs, freelancers, teachers, farmers, private employees, and students, with 6 participants not currently employed. The participants were from 3 major tribes in South Sulawesi: 7 participants from the Bugis tribe, 6 from the Makassar tribe, and 2 from the Toraja tribe. All participants had undergone hemodialysis therapy for a period ranging from 2 to 8 years, with the majority receiving therapy twice per week.

Participants underwent PCR swab tests between 3 and 20 times. Researchers made three contacts with each participant, either through face-to-face interviews or telephone calls, due to the COVID-19 pandemic (as shown in Table 1).

**Table 1.** Participant characteristics.

Participants	Length of time undergoing hemodialysis (years)	Schedule undergoing hemodialysis/week	Frequency of PCR swabs	Number of meetings
1	5	2 times	14 times	3 times
2	6	2 times	15 times	3 times
3	3	2 times	15 times	3 times
4	2	2 times	6 times	3 times
5	2	2 times	10 times	3 times
6	4	2 times	10 times	3 times
7	7	2 times	9 times	3 times
8	3	2 times	11 times	3 times
9	2	2 times	11 times	3 times
10	4	2 times	6 times	3 times
11	8	2 times	9 times	3 times
12	3	2 times	10 times	3 times
13	4	2 times	20 times	3 times
14	5	2 times	3 times	3 times
15	2	2 times	9 times	3 times

### Thematic analysis results

The experiences of end-stage renal disease patients undergoing hemodialysis in the era of the COVID-19 pandemic have yielded three overarching themes. These themes encompass the following: (1) the emergence of diverse responses at the onset of the pandemic, (2) the emergence of various impacts throughout the pandemic, and (3) the development of coping strategies during the pandemic.

***Theme 1: The emergence of diverse responses at the onset of the pandemic***

The research team identified the initial theme through an analysis of participant responses regarding their experiences with hemodialysis during the early stages of the pandemic. The findings revealed a range of negative reactions, which were grouped under the sub-theme “emotional response and behaviour response.”

*Emotional response*

At the outset of the pandemic, participants described a range of negative emotional responses. At the outset of the COVID-19 pandemic, individuals undergoing hemodialysis expressed concerns, apprehension, and distrust. Anxiety was reported by nearly all participants at the onset of the pandemic. The majority of participants expressed concern that they might encounter difficulties in accessing hemodialysis in the event of a positive test result for SARS-CoV-2. Additionally, the majority of participants expressed concern about developing symptoms similar to those observed in patients with COVID-19 at the onset of the pandemic. It is not uncommon for patients with chronic kidney failure undergoing hemodialysis to present with symptoms such as cough, shortness of breath, flu-like symptoms, and fever. Some participants indicated that they experienced feelings of anxiety because of being suspected by other patients or the nurses, that they may be carrying the SARS-CoV-2 virus.

*“This swab keeps making me scared because this also keeps my blood pressure rising” (P2).*

*“I am afraid because it will be difficult for me later. Finding hemodialysis schedules and entering isolation are problematic. It is hard if we get COVID-19” (P3).*

*“Had I been provided with the swab, I would have experienced a sense of unease and anxiety tend to ruminate on issues, which can precipitate a state of physical illness” (P4)*

*“We have a disease and are prone to be affected. That is why there is a disease that we carry. We were nervous about receiving the swab results” (P5).*

*“Frequently, I experienced feelings of anger and frustration. However, I refrained from expressing my emotions towards other patients. At most, I informed the nurse to request a swab test, as I was concerned about the potential risk of contracting a disease in this environment” (P13).*

*“I feared that I would not be provided with a hemodialysis machine, given the limited availability of such devices for use in patients with COVID-19” (P14).*

Furthermore, some participants articulated apprehension regarding the potential for testing positive for COVID-19 infection due to their classification as a high-risk group. This concern arose because patients with chronic kidney disease have an immune system that is compromised, rendering them more susceptible to infection by COVID-19, as expressed by the participants:

*“We are at higher risk because we have a pre-existing condition. That is why we are anxious” (P5).*

*“The result of the swab should not be positive because my fear is overwhelming. It is not just that we (hemodialysis patients) have kidney disease, but the doctors have said we are more susceptible, more at risk of contracting COVID-19” (P6).*

*“What worries me is the isolation room because no visitors are allowed. I feel bad because I cannot walk or do anything on my own, so I am worried about how I will manage inside if no one can visit” (P9).*

*Behavioural response*

In the initial stages of the global pandemic caused by COVID-19, individuals exhibited a range of behavioural responses to concerns, fears, and suspicions. Such behaviors include adhering to hemodialysis regimens, concealing one’s health status, and disregarding social etiquette. This is particularly evident among patients with terminal chronic renal failure who undergo hemodialysis therapy to sustain their survival. From the start of the pandemic, participants expressed concern and apprehension about COVID-19. Despite this, all participants made efforts to continue with their hemodialysis treatment.

*“I’m concerned because my throat is itchy and I’ve been told that coughing is a symptom of corona. If I can handle it myself, I’d rather do so and not have to tell anyone” (P2)*

*“what else can I do? This is my fate; I have to go to dialysis if I want to survive” (P15)*

Participants chose to hide their own or their family’s health status due to fear of being stigmatized as having COVID-19 or being in contact with someone who had it. For example, one participant mentioned:

*“I hope it’s just a regular cough because of an itchy throat and not labelled as COVID-19. If it’s something we can treat, let’s not jump to conclusions” (P2)*

*“I hid my husband from the nurse because I was afraid that my husband would be suspected, even though I was the one who was sick, my husband was not” (P3)*

Another behavioural response observed was a disregard for social norms and etiquette. Some participants exhibited a tendency to disengage from social interactions because they were excessively afraid of contracting COVID-19.

*“Close friends now keep their distance, telling me to ‘stay away.’ They weren’t like this before, but now they are. I’d rather this than risk catching COVID-19” (P2).*

*“I’m cautious about who comes into my house. I’d rather offend someone than take the risk. Neighbors who used to visit now stay away because I get upset with them. Let them be offended; I’d rather be safe” (P3).*

***Theme 2: Various impacts***

The COVID-19 pandemic has had a significant impact on various aspects of the participants’ lives as patients with end-stage renal disease undergoing hemodialysis. These impacts are summarized in the sub-themes of physical, psychological, social, and economic effects, as well as the implementation of health protocols experienced by participants during the pandemic.

*Physical impact*

The majority of participants reported experiencing physical effects, including weight gain during interdialytic periods, shortness of breath, elevated blood pressure, pain, discomfort, and bleeding. The most frequently reported issue was interdialytic weight gain. As a result of the pandemic, patients’ hemodialysis schedules were postponed if their swab results were unavailable. In some cases, it was found that the results of the swab were received 1-2 days after swabbing process, due to limitations in resources. In addition, some patients’ schedules have been modified as a result of the hemodialysis unit’s efforts to optimize the hemodialysis schedule. Shortness of breath represents a physical impact experienced by participants as a consequence of delays or alterations to their hemodialysis schedules. An increase in blood pressure was found as a physical impact experienced by hemodialysis patients during COVID-19 pandemic due to anxiety about swab results. Some participants exhibited considerable anxiety regarding the results. The swab yielded a positive result, which was accompanied by an increase in blood pressure. The subsequent physical impact is pain. The mandatory swab obligations carried out by participants are a cause of pain, which is necessary for them to continue undergoing the hemodialysis process. Although participants reported discomfort and pain related to repeated swab testing, they emphasized that this was not as severe as the pain and complications caused by fluid overload, which represents a more critical physical burden for hemodialysis patients.

Many patients complained of weight gain during interdialysis due to the delay of hemodiakysis schedule during the COVID-19 pandemic.

*“My body feels heavy. They usually remove three litres of fluid, but now they only remove two or two and a half litres during the four-hour sessions” (P1).*

*“My stomach is bloated, and my legs are swollen. They removed three litres before, but now it is only two” (P2).*

Another physical impact reported by the participants was difficulty breathing, which was often the result of postponed or rescheduled hemodialysis sessions.

*“It gets really bad; I feel breathless, and it gets worse if I miss a session of hemodialysis” (P9).*

*“Yes, I feel breathless, especially if I have not had hemodialysis for a while” (P12).*

The subsequent physical impact observed was an increase in blood pressure due to anxiety over swab test results. Several participants experienced significant anxiety at the prospect of testing positive for COVID-19, which in turn led to elevated blood pressure. The following are quotes from some participants:

*“The swab test is what scares me, and it causes my blood pressure to rise. It is always on my mind, especially when waiting for the results—my results are coming out tomorrow, and I am so anxious” (P2).*

*“After a swab, I feel uneasy. Before the pandemic, my blood pressure was normal. Now it often spikes. It is still high and rarely goes down” (P4).*

The other physical impact is pain. This is a direct consequence of the mandatory swab, which participants must carry out in order to continue undergoing the hemodialysis process. The process of taking samples for swab examination, which occurs every 2-3 weeks, causes pain for some participants. The following statements from several participants illustrate this point.

*“The swab every 14 days hurts my nose” (P2).*

*“This swab is really painful. It is only a matter of time before I start screaming” (P9).*

*Psychological impact*

The COVID-19 pandemic has also had a significant psychological impact on patients undergoing hemodialysis. The necessity of awaiting the results of the swab and the obligation to undergo continuous swabs has resulted in the patient experiencing disturbances to their sleep patterns, a sense of being a burden, and a perception of being under constant observation during the course of this pandemic. The majority of participants reported experiencing disturbances in their sleep patterns during the pandemic. However, during the current pandemic, these problems have been exacerbated due to concerns about the results of the swab. The majority of participants reported feeling burdened by the obligation to undergo swab tests. The obligation to undergo swabbing every 2-3 weeks necessitates that participants visit a hospital outside of their scheduled hemodialysis appointment and other scheduled examinations. The process of swabbing is quite intricate and time-consuming. The extended period is a significant burden for participants, particularly given their history of complications related to declining kidney function. One participant also reported a psychological impact resulting from the constant observation by nurses.

Many participants stated that during the pandemic, their sleep patterns were disrupted after knowing their swab result.

*“When it is close to the following swab schedule, I often have trouble sleeping. The stress and anxiety that come with the condition make it difficult for me to get the rest I need. As my stress levels rise, I find it even harder to sleep” (P8).*

*“I’m so scared, I’m really worried. I hardly ever sleep at night. I’m always thinking about when the phone will ring, telling me I’m positive COVID-19” (P11).*

Another psychological impact was the sense of being overwhelmed by the frequent swab tests. The necessity of attending the hospital outside of their regular hemodialysis schedule introduced an additional source of stress to an already challenging situation. The process of scheduling and undergoing the tests was found to be overwhelming by the participants, as evidenced by the following description:

*“It’s such a burden to have to do the swab every 21 days. The whole process—the route, the queue, registering, then seeing the doctor the next day, and so on—it’s exhausting” (P12).*

Another participant echoed this statement:

*“The process of undergoing a swab is quite exhausting, I must waiting in a queue for a long time, and I feel unsatisfied because the distance between screening corner which located at the emergency room and the swab room is quite far. This cycle occurs every two weeks, and the fatigue associated with it persists” (P13).*

One participant believed that being observed continuosly by the nurse constituted a psychological influence that also occurred.

*“It feels like I’m always being watched during this pandemic. The nurse keep asking when I’ll have to swab again” (P9).*

*Social impact*

Additionally, several participants reported experiencing social impacts as a result of the pandemic. A reduction in social interaction and a sense of ostracism were among the social impacts experienced by participants in the hemodialysis unit. Other social impacts include instances of ostracism. Some participants have been avoided by their families and neighbours during the pandemic due to the assumption that they had contracted the COVID-19 virus from the hospital.

*“Upon arrival at the hemodialysis unit, I found it necessary to limit my interactions with others. I maintained a distance and focused on personal hygiene, such as cleaning my bed. The atmosphere was noticeably different from what I had experienced previously” (P2).*

The pandemic also had a profound social impact on participants, particularly in terms of social isolation.

*“We feel like we’re at a distance from our friends. Before, we could forget about our illness by spending time with friends, but now we have to keep our distance” (P7).*

Others were shunning another social impact. Some participants reported being avoided by their neighbours and family members due to fears that they might bring COVID-19 home from the hospital.

*“It’s frustrating when I visit neighbours, and they immediately leave when I arrive. I tell them I don’t have the virus, but they don’t listen. I have a swab test result every two weeks, but they ignore me” (P5).*

*“People think the hospital is full of COVID-19, so when I come home from the hospital, they avoid me” (P7).*

*Economic impact*

Additionally, several participants reported experiencing the economic consequences of the global pandemic caused by COVID-19 pandemic. The cost of living has risen for those who require hemodialysis therapy. The obligation to undergo swabbing procedures has resulted in an increase in the number of visits to the hospital by participants, necessitating the expenditure of funds on transportation. Furthermore, participants are required to bear additional costs associated with the procurement of personal protective equipment, including masks.

*“The issue is that wearing a mask is quite difficult. I believe masks are not meant to be used twice, so we have to replace them. If we’re on a tight budget, we have to use them twice. We used the first mask 2-3 times. It is much work. That’s why my husband is back in his hometown to earn money for hospital expenses” (P7).*

***Theme 3: Coping mechanisms developed***

*Adaptive coping*

Adaptive coping is the most prevalent coping strategy individuals employ in response to challenges encountered during the ongoing pandemic. Other coping adaptations employed by participants include spiritual strengthening.

*“I pray to God every day and night. Hopefully, we won’t get infected with COVID-19” (P11).*

*“We are sick, and the swabs make us feel worse, so we keep praying that these swabs will stop soon" (7).*

Some participants attempted to maintain a positive outlook despite the negative emotions they experienced during the pandemic.

*“I worry sometimes, but I know my immunity is good, so I just stay positive” (P4).*

*“I try to stay calm and face everything head-on. This is not something that comes easily to everyone, so I tell my friends that maybe we’re being tested. Alhamdulillah, it gives me strength, so I stay motivated” (P5).*

Another adaptive coping strategy was dietary change. Some participants modified their diets to forestall any complications before undergoing swab tests.

*“I’ve been trying to cut back on my fluid intake and improve my diet. I make sure to eat well every time I have a swab coming up” (P5).*

*“I changed my diet because I used to eat oily and fried foods. Since the pandemic, I avoid them. I don’t eat ice cream anymore. I’m afraid of getting short of breath before the swab, so I limit my diet” (P7).*

Furthermore, participants demonstrated an ability to adapt to the revised schedule and the necessity of utilizing personal protective equipment (PPE) during hemodialysis.

*“When I first started, I was always late for my hemodialysis schedule in the morning, but after a couple of weeks, I got into a routine and stopped being late in the morning” (P1).*

*“I have become accustomed to the new protocol. Initially, I found it unusual to be instructed to wear a mask, but I have since made it a routine practice. It is now unusual for me not to wear a mask at the hospital” (P14).*

*Maladaptive coping*

While some participants employed adaptive coping strategies in response to the various challenges and difficulties encountered during the ongoing pandemic, others resorted to maladaptive coping mechanisms to deal with the pandemic’s multifaceted impacts. Some participants treat themselves if they felt having similar to COVID-19 symptoms, employed excessive prevention and withdrawal mechanisms as a means of adapting to the conditions of the pandemic.

*“When I got fever, cough or shortness of breath, I took vitamin C, folic acid and played sports despite the rain. I also drank honey and boiled ginger, which high in potassium, but I do not care. The main thing is that it is clear: do not be exposed to COVID-19” (P3).*

*“I slept with a mask on. My child asked why I wore a mask to sleep. I said it was because I was afraid of COVID-19. I also bought vitamins from the pharmacy because I was scared.” (P6).*

Another maladaptive coping strategy observed was social withdrawal during hemodialysis. Some participants elected to withdraw from social interactions in order to circumvent the stigma associated with their condition. As individuals with end-stage renal disease, they were required to undergo hemodialysis two to three times per week at the hospital. However, the stigma associated with COVID-19 prompted them to avoid contact with others.

*“I do not want to be identified as a patient with COVID-19. When I am in the hospital, I do not talk to or touch other people” (P4).*

*“I’d rather stay silent than explain myself. If people misunderstand, I accept it. I can’t change what they think” (P10).*

## Discussion

Stress during an infectious disease outbreak such as fear and worry about health problems also can be worsening of chronic conditions, can significantly impact individuals ( CDC, 2020). This aligns with research conducted by Xia et al. (2020), which highlights the high level of psychological distress experienced by hemodialysis patients at the start of the pandemic. This distress was largely due to the frequent visits required for dialysis therapy and concerns about transportation. Supporting this, Guerraoui et al. (2021) found that anxiety and depression were prevalent among hemodialysis patients in the early days of the pandemic, driven by fears of contracting COVID-19, the high incidence of the virus, and concerns about transmitting it to family members.

At the onset of the pandemic, most participants expressed concern about contracting COVID-19, which created difficulties in accessing hospital services. Hemodialysis schedules were frequently overcrowded, and the limited number of healthcare personnel posed challenges in preparing dialysis machines for patients who tested positive for COVID-19. In addition, some participants reported fear of being stigmatized or ostracized if they became infected. Infectious diseases are often associated with stigma, leading to discrimination against affected individuals ( Abdelhafiz & Alorabi, 2020). Pandemics such as COVID-19 can heighten fear and anxiety, further contributing to social stigma and avoidance behaviors ( Turner-Musa et al., 2020). Moreover, hemodialysis patients commonly experience symptoms such as fever, cough, and shortness of breath—symptoms that overlap with those of COVID-19 ( IRR, 2018). This overlap led to increased suspicion and mistrust among patients toward one another.

Previous studies shown that patients experienced feelings of worry and fear that hemodialysis can make them non-compliant with medication therapy, including non-compliance in undergoing hemodialysis therapy ( Kimmel, 2001; Kimmel & Peterson, 2005). However, the findings of this study present a different perspective. Despite experiencing worry, fear, and suspicion during the early stages of the COVID-19 pandemic, most hemodialysis patients reported maintaining adherence to their hemodialysis sessions. Participants emphasized that the risks associated with missing a session were perceived as more serious than their concerns about COVID-19 infection. Hemodialysis was considered essential to sustaining their quality of life and preventing further complications that could arise from non-compliance with treatment.

Excessive emotional responses can lead to negative behaviors. COVID-19, as a new infectious disease, has triggered heightened anxiety and fear, which can result in dangerous behaviors ( Ho et al., 2020). This study found that the worry, fear, and suspicion experienced by participants during the early days of the pandemic led to several negative behaviors, such as concealing their health status and disregarding social etiquette. Participants chose to hide their health status, particularly when they exhibited symptoms similar to those of COVID-19 or had a history of contact with infected family members. This was done to avoid being referred to isolation rooms or undergoing COVID-19 screenings, which could cause delays in their hemodialysis schedules. As a result, participants with symptoms like fever, cough, and shortness of breath chose to self-medicate. This behavior aligns with research by Villa et al. (2020), which suggests that stigma may discourage individuals from acknowledging potential COVID-19 symptoms, leading them to hide their symptoms to avoid social pressure. This causes patients with mild symptoms to forgo treatment and behave normally to prevent drawing attention to their condition.

At the onset of a crisis or pandemic, individuals and communities typically seek information about the situation. However, when official sources or trusted news are lacking, people often turn to social media or other unreliable sources, which can fuel negative emotional responses and amplify fear, uncertainty, and distorted risk perceptions ( Torales et al., 2020). Research by Wang et al. (2020a) highlights that the continuous dissemination of accurate and updated health information from trusted authorities can reduce anxiety, stress, and depressive symptoms. Therefore, the role of nurses as educators is essential.

The COVID-19 pandemic has had significant impacts on physical health, mental health, social life, and the economy. Patients with chronic diseases, in particular, have experienced substantial health challenges due to delays or difficulties in accessing healthcare services during the pandemic ( Singh et al., 2021). The World Health Organization ( WHO, 2020) reported that more than half of the countries affected by COVID-19 experienced disruptions in care services for patients with chronic conditions. An especially ironic situation arose for hemodialysis patients, who require routine or long-term treatment. This study found that the COVID-19 pandemic negatively impacted the physical health of kidney failure patients undergoing hemodialysis, primarily due to delays and changes in their treatment schedules. In addition to the physical health impacts, the pandemic has also had significant mental health consequences ( Pfefferbaum & North, 2020).

The mental health and psychosocial impacts of the COVID-19 pandemic may be more severe among individuals with chronic diseases, as they are at higher risk of severe complications if infected, which increases perceived stress and worsens overall health outcomes ( WHO, 2020). In this study, participants reported various psychological impacts, including high stress related to the possibility of testing positive for COVID-19, which contributed to sleep disturbances, increased blood pressure, feelings of burden, and social rejection. The pandemic has long-term consequences for the physical and mental health of hemodialysis patients. At the time of the initial outbreak, the World Health Organization (WHO) actively sought to control and reduce the pandemic’s impact by identifying, testing, and treating infected patients, as well as developing drugs, vaccines, and treatment protocols. However, success in overcoming the pandemic could not be guaranteed due to its unpredictability ( Kumar & Nayar, 2020). Chronic disease sufferers also experienced lifestyle disruptions, including reduced physical activity, sleep problems, and heightened stress and mental health concerns, which required further management ( Kendzerska et al., 2021). Similar findings were reported in other studies, which demonstrated elevated levels of anxiety and depression among hemodialysis patients during the pandemic ( Kosunalp & Kavurmaci, 2023; Nadort et al., 2022). Research by Yang et al. (2021) also highlighted the relationship between psychological distress and reduced quality of life in this population. These results underscore the urgent need for psychosocial and psychotherapeutic interventions to support mental health and improve the quality of life of hemodialysis patients during and beyond the pandemic.

Hemodialysis patients also experience social impacts. Most participants reported a decrease in social interaction among fellow hemodialysis patients. Research by Yang et al. (2021) also highlighted social dysfunction in patients during this time. The demand for hemodialysis has significantly increased since the onset of the pandemic. Therefore, hemodialysis nurses could consider creating innovative, engaging, and easily accessible chat groups for hemodialysis patients and their families.

The COVID-19 pandemic increases the cost burden on hemodialysis patients, mainly by supplying funds for purchasing personal protective equipment. Apart from that, swabs and other examinations are mandatory. The pandemic caused an increase in the frequency of hospital visits, this has increased the amount of personal protective equipment that must be prepared and increased costs for transportation. Research conducted by Lee et al. (2020) also shows that 90% of patients in hemodialysis are concerned about the economic impact and difficulties of finances during the COVID-19 pandemic. Adjusting swab examination schedules and other examinations with hemodialysis schedules may be a dialysis unit consideration. Reducing the frequency of going to the hospital can reduce transportation costs. Hemodialysis patients can simultaneously reduce the risk of exposure to COVID-19 both during travel and in the hospital. Beyond the costs of personal protective equipment and swab tests, participants also faced broader economic challenges, including reduced income, loss of employment benefits, and increased difficulty in maintaining appropriate dietary requirements during the pandemic.

Participants in this research also developed various coping strategies to deal with the various stressors experienced in the COVID-19 pandemic. During the pandemic, the majority of participants employed adaptive coping strategies such as spiritual strengthening, positive thinking, and efforts to accept and acclimatize to their circumstances. Several previous studies also show that there are efforts to implement adaptive coping strategies in facing the COVID-19 pandemic. Some of the examples were relying on increased social support ( Cao et al., 2020) and adopting preventive measures to offset or minimize health risks and finances brought about by COVID-19 ( Wang et al., 2020a). Some participants described spiritual practices as an important coping mechanism that helped them adapt to uncertainty and stress during the pandemic. In addition, several participants noted challenges in balancing treatment with family responsibilities, which at times were perceived as an added burden alongside their illness. These findings highlight the need for dialysis nurses to provide targeted psychosocial support that recognizes both spiritual resources and family-related stressors, thereby promoting resilience and overall well-being among hemodialysis patients.

Maladaptive coping refers to coping strategies associated with poor mental health outcomes and symptoms of higher levels of psychopathology, such as avoidance and emotional suppression ( Compas et al., 2020). In this research, several participants also developed maladaptive coping during the pandemic, include taking excessive preventive actions and withdrawing hemodialysis to face various stressors during the pandemic. Several previous studies identified factors of demographics as a potential risk of implementing maladaptive coping strategies in dealing with the COVID-19 pandemic, such as age, gender, race, socioeconomic status, and the burden of being parents ( Atchison et al., 2021; Clay & Parker, 2020). However, this study did not find a relationship between demographic data and participants’ maladaptive coping strategies. Therefore, differences in the participants’ personalities can be a factor in using coping strategies in this research. This is relate to the research conducted by Bacon & Corr (2020) and Carvalho et al. (2020) state that individual personality functions influence their adaptive and coping strategies related to health problems during the COVID-19 pandemic.

How individuals view and deal with situations caused by COVID-19 can significantly affect their mental and physical health and their willingness to engage in protective behaviors (Brailovskaia & Margraf, 2020). According to Roy’s theory, various stimuli related to the pandemic, such as social, psychological, and physical stressors, influence how hemodialysis patients adapt to their disease conditions ( Roy, 2009). The findings of this study showed that most participants attempted to adapt by maintaining adherence to hemodialysis therapy despite fear of COVID-19 infection and by adopting adaptive coping strategies such as strengthening religious practices and seeking family support. However, some participants demonstrated maladaptive strategies, including avoidance and withdrawal, which could worsen their condition. Differences in personality traits also contributed to variations in coping responses, consistent with previous evidence that personality influences coping during health crises ( Bacon & Corr, 2020; Carvalho et al., 2020). Similar to our results, Dehghan et al. (2021) found that mindfulness and coping strategies were strongly associated with anxiety, stress, and mental health among hemodialysis patients during the pandemic. These findings highlight the importance of reinforcing adaptive coping skills and providing professional support to reduce maladaptive responses. Individuals with positive coping strategies usually report fewer symptoms of anxiety and stress ( Moccia et al., 2020; Wang et al., 2020b).

Dialysis nurses have a vital role in ensuring holistic care that addresses not only the physical but also the psychological and social needs of hemodialysis patients. These results highlight the need for dialysis nurses to actively support patients’ mental health, which has also been emphasized in recent studies examining depression, anxiety, and quality of life among hemodialysis patients during the COVID-19 pandemic ( Nadort et al., 2022; Kosunalp & Kavurmaci, 2023). Moreover, dialysis nurses are expected to educate patients about effective coping strategies and create supportive environments that reduce psychological distress and improve adherence to treatment. Innovative interventions, such as the development of easily accessible communication platforms and peer-support initiatives, may also help patients and families manage stress more effectively during health crises. By providing continuous psychosocial support, dialysis nurses can play a key role in promoting resilience and improving the overall quality of life of patients undergoing hemodialysis.

## Conclusion

Results of research on end-stage renal disease patients undergoing hemodialysis in the era of the COVID-19 pandemic illustrate three main themes, consist of (1) the emergence of various responses to the beginning of the pandemic both through emotional and behavioral responses, (2) the emergence of various impacts during a pandemic such as physical, psychological, social and economic impacts and (3) the existence of coping strategies that were built during the pandemic, namely adaptive and maladaptive coping.

These findings indicate that hemodialysis patients require continuous support from healthcare providers to address the multifaceted challenges posed by the pandemic. Strengthening psychosocial interventions, such as counseling, stress management, and peer-support groups, alongside ensuring adherence to physical care, will be essential. Furthermore, the results underscore the importance of continuous professional development for dialysis nurses in delivering holistic, patient-centered care and enhancing resilience among patients during current and future health crises.

### Limitations

Due to the COVID-19 pandemic, interviews were limited to face-to-face sessions with 4 participants and phone interviews with 11 participants, based on their comfort levels. Health protocols during face-to-face interviews sometimes led to unclear responses, requiring follow-up questions, which may have affected the depth of the data. Additionally, the reliance on phone interviews limited the observation of non-verbal cues, potentially impacting the richness of the insights. The study’s findings are also based on a specific sample from two hospitals in Indonesia, which may limit their generalizability to other populations and contexts. During the period when this study was conducted, compared with the current context, the COVID-19 pandemic as the issue of this research, has changed significantly. Because it’s no longer as severe as it was. However, the findings from this research provide valuable new insights into the needs of hemodialysis patients when dealing with challenging condition.

### Implications of this study

**Clinical practice implications**

Strengthening psychosocial interventions, including stress management and peer-support groups, should be prioritized in hemodialysis units to enhance patient resilience during future health crises. Healthcare providers, especially dialysis nurses, should deliver holistic care and provide sufficient information about the pandemic to address both physical and psychological needs, thereby reducing patients’ anxiety and stress. Furthermore, the findings of this study highlight the importance of enhancing continuing professional development programs for dialysis nurses to strengthen their capacity in managing patients with chronic disease during pandemic situations.

**Health policy implications**

Health policies should prioritize preparedness for future pandemics by ensuring flexible care schedules and maintaining access to essential treatments, minimizing disruptions to patient care in times of crisis. Continuous evaluation of healthcare systems’ resilience is necessary to protect vulnerable patients in any future public health emergencies.

**Research implications**

This study is a valuable reference for developing bio-psycho-social interventions tailored to chronic kidney failure patients receiving hemodialysis, particularly during pandemic conditions. Further studies are needed to explore factors influencing patients’ coping strategies and to evaluate the long-term effects of the pandemic on the quality of life of dialysis patients.

## Ethical considerations 

Research ethics were ensured through the approval of the Faculty of Nursing Ethics Committee at the University of Indonesia, documented under ethical approval number: SK-15/UN2.F12.DI.2.1/ETIK 2021, dated February 3rd, 2021. The researchers provided explanations regarding the procedures, objectives, benefits, potential risks, and the rights and responsibilities of participants through a research information sheet. Participation was voluntary, as indicated by the signing of informed consent. Participants were also assured that their involvement was voluntary and were informed of their right to withdraw from the study at any time without facing any penalties.

## Data Availability

The interview transcripts cannot be publicly shared because the data contain sensitive information that could potentially identify the participants. The restriction is a guideline set by the ethics committee and included in the informed consent that participants have agreed to. Access to the data is restricted to protect participant confidentiality. Any data access requests must be reviewed and approved by the authors and will only be granted under conditions that ensure participant anonymity. All transcripts are in Bahasa. Those interested in reading the summary report, including quotes, may contact the corresponding author ( dewiyantitodinguh@gmail.com) for translations of the requested sections. Fighshare: COVID-19 pandemic: A Qualitative Study. figshare. Journal contribution, https://doi.org/10.6084/m9.figshare.27310995.v2 ( Toding, 2024). This project contains the following extended data:
a.
**Research explanation**: This section provides participants with details on the study’s objectives, methods, potential benefits, risks, and their rights. It also explains data usage and protection measures, along with how participants can ask questions or choose to withdraw from the study if necessary b.
**Informed consent**: this is the voluntary consent participants provide after being fully briefed on the study’s details, as outlined in the research explanation provided to them. c.
**Reporting guidelines**: we used COREQ checklist Data are available under the terms of the Creative Commons Attribution 4.0 International license (CC-BY 4.0).
