# Supplementary material for: Exploring the experiences of the hemodialysis patients during the COVID-19 pandemic: A qualitative study
Source: F1000Res. 2025 Sep 22;13:1404. Originally published 2024 Nov 21. [Version 2] doi: 10.12688/f1000research.158482.2 (PMC12759275; doi:10.12688/f1000research.158482.2)
Supplement: Peer review history

# Reviewer report: Reviewer response for version 2

**DOI:** 10.5256/f1000research.188121.r439650

**Published:** 2026-01-15

**Authors:** Salia Virxinia Pol Heres

**Affiliations:** 1 Universitario Virgen Macarena, Sevilla, Spain; 2 St George's University Hospitals NHS Foundation Trust, London, England, UK

**Summary of the article**

 This qualitative study explores the experiences of patients with end-stage renal disease receiving in-centre hemodialysis during the COVID-19 pandemic in Indonesia. Using in-depth interviews and guided by Roy’s Adaptation Model, the authors describe emotional, physical, social, and economic impacts of the pandemic, as well as adaptive and maladaptive coping strategies. The study focuses on a vulnerable population and addresses an important and timely clinical issue.

**Overall assessment**

 I found this to be a well-conducted and clearly presented qualitative study. The methodology is appropriate, the analysis is coherent, and the use of participant quotations effectively supports the identified themes. The findings are clinically meaningful and relevant to nursing and multidisciplinary renal care, particularly in the context of public health crises.

 The manuscript is balanced, thoughtful, and provides valuable insight into patients’ lived experiences. While there are always small areas where clarity could be further improved, these do not detract from the overall quality or scientific soundness of the work.

**Optional suggestions**

- A slightly more explicit linkage between the findings and Roy’s Adaptation Model may help reinforce the theoretical framing, though the current presentation is acceptable.
- Minor language editing could further improve flow in a few sections, but this is not essential.

**Conclusion**

 This article makes a meaningful contribution to the literature on dialysis care during the COVID-19 pandemic and highlights important psychosocial aspects of patient experience. I support indexing of the manuscript in its current form.

Is the work clearly and accurately presented and does it cite the current literature?

Yes

If applicable, is the statistical analysis and its interpretation appropriate?

Yes

Are all the source data underlying the results available to ensure full reproducibility?

Yes

Is the study design appropriate and is the work technically sound?

Yes

Are the conclusions drawn adequately supported by the results?

Yes

Are sufficient details of methods and analysis provided to allow replication by others?

Yes

Reviewer Expertise:

Renal medicine, hemodialysis, cardiorrenal medicine

I confirm that I have read this submission and believe that I have an appropriate level of expertise to confirm that it is of an acceptable scientific standard.

# Reviewer report: Reviewer response for version 2

**DOI:** 10.5256/f1000research.188121.r436381

**Published:** 2026-01-02

**Authors:** Yafi Sabila Rosyad

**Affiliations:** 1 Nursing, Universitas Bhakti Husada Indonesia, Kuningan, Indonesia; 2 Mental health nursing, Putrajaya, Malaysia

The manuscript presents a qualitative exploration of the experiences of hemodialysis patients during the COVID-19 pandemic, addressing an important clinical and psychosocial issue within a vulnerable population. Overall, the study demonstrates strong potential, especially because it captures patient experiences at a critical moment when healthcare systems were under extreme strain. The use of a qualitative descriptive design supported by Roy’s Adaptation Model is appropriate for the study’s aims and contributes theoretical grounding to the analysis. The manuscript is generally well structured and detailed; however, several areas require refinement to enhance clarity, methodological rigor, and the interpretive depth expected by high-impact journals.

 The introduction is comprehensive and provides sufficient background on the vulnerability of hemodialysis patients in the context of COVID-19. However, it is somewhat lengthy and could be more sharply focused on the specific knowledge gap that this study aims to address. The current narrative reviews many aspects of COVID-19 risk and dialysis care, but the justification for why a qualitative descriptive inquiry is needed—particularly in the Indonesian context—emerges later than expected. A more concise and problem-focused introduction would strengthen the argument for the study’s relevance and novelty.

 The methods section is rich in procedural detail and shows improvement in version 2, especially with the inclusion of Lincoln and Guba’s criteria for rigor. The justification for the qualitative descriptive design is also clearer. Still, several aspects could benefit from further clarification. The description of data saturation remains general; adding explicit examples of how saturation was recognized at each site would help readers assess the adequacy of the sampling strategy. The reliance on telephone interviews for the majority of participants is understandable given pandemic restrictions, but the manuscript should discuss more explicitly how this modality may have affected rapport building, depth of responses, and observation of nonverbal cues. The description of the interviewer’s expertise is appreciated, but reflexivity—how the interviewer’s clinical background may have influenced data collection and interpretation—requires deeper reflection.

 The findings are organized into three main themes that are logical and supported by rich participant quotes. The theme on emotional and behavioral responses at the onset of the pandemic is particularly compelling and aligns with global reports of fear, stigma, and uncertainty among chronic disease populations. The manuscript effectively captures the complexities of patients’ experiences, including their fears of infection, difficulties navigating hospital routines, and emotional burden of repeated swab testing. However, some subthemes appear repetitive, and the manuscript would benefit from more synthesis rather than presenting long lists of examples. In several cases, quotes could be shortened or curated more strategically to maintain narrative flow while preserving analytic depth.

 The second theme, highlighting the physical, psychological, social, and economic impacts of the pandemic, is comprehensive but occasionally becomes descriptive rather than analytical. While the lived experiences are vividly portrayed, the manuscript could deepen the interpretation by engaging more critically with existing literature—not only comparing findings but also explaining divergences or unique cultural patterns identified in this population. Given the relevance of socioeconomic challenges in Indonesia, the economic impact deserves stronger theoretical or policy-oriented interpretation.

 The third theme on coping strategies is well articulated, distinguishing between adaptive and maladaptive coping. The integration with Roy’s Adaptation Model becomes more visible here, but the theoretical connections could still be strengthened. For instance, the discussion could more explicitly show how specific stimuli (contextual, focal, residual) shaped adaptive or maladaptive modes of response among participants. In particular, there is an opportunity to analyze how spirituality, family dynamics, and cultural expectations contributed to adaptive mechanisms—a crucial dimension in Indonesian healthcare settings.

 The discussion section incorporates a wide range of recent literature and contextualizes the findings well. Nonetheless, the section could be significantly more concise. Some paragraphs repeat earlier explanations, and multiple citations reiterate well-established knowledge. A tighter focus on the study’s unique contributions—such as the interplay between COVID-19 protocols and dialysis access, or the role of stigma in shaping patient behavior—would help elevate the manuscript’s impact. Additionally, the discussion occasionally shifts from interpretation to general commentary about the pandemic; maintaining analytical discipline would improve cohesion.

 The conclusion is consistent with the findings, but it would benefit from more actionable recommendations for clinical practice and policy development. The current implications section does offer concrete suggestions, especially regarding psychosocial support and professional development for dialysis nurses, but these could be integrated more directly into the concluding remarks to emphasize their significance.

 Ethical considerations and data availability statements are clearly presented and align with best practices. However, the restriction on data sharing, while understandable, may raise questions in journals committed to open science. Providing a detailed justification—possibly including anonymization challenges—would help address this concern.

 Overall, the manuscript is well developed and showcases meaningful findings that could contribute to nursing practice and public health management during pandemics. However, improvements in conciseness, analytical depth, and theoretical integration are needed. With careful refinement, the paper has strong potential for indexing in a reputable Scopus-indexed journal.

Is the work clearly and accurately presented and does it cite the current literature?

Yes

If applicable, is the statistical analysis and its interpretation appropriate?

Partly

Are all the source data underlying the results available to ensure full reproducibility?

Yes

Is the study design appropriate and is the work technically sound?

Partly

Are the conclusions drawn adequately supported by the results?

Yes

Are sufficient details of methods and analysis provided to allow replication by others?

No

Reviewer Expertise:

Nursing, Mental health nursing, qualitative

I confirm that I have read this submission and believe that I have an appropriate level of expertise to confirm that it is of an acceptable scientific standard, however I have significant reservations, as outlined above.

# Reviewer report: Reviewer response for version 2

**DOI:** 10.5256/f1000research.188121.r416416

**Published:** 2025-09-24

**Authors:** Mehtap Kavurmaci

**Affiliations:** 1 Nursing Faculty, Department of Internal Medicine Nursing, Atatürk University, Erzurum, Turkey

The authors have satisfactorily addressed the suggested revisions. The manuscript is suitable for indexing in its current form.

Is the work clearly and accurately presented and does it cite the current literature?

Partly

If applicable, is the statistical analysis and its interpretation appropriate?

Yes

Are all the source data underlying the results available to ensure full reproducibility?

Yes

Is the study design appropriate and is the work technically sound?

Partly

Are the conclusions drawn adequately supported by the results?

Yes

Are sufficient details of methods and analysis provided to allow replication by others?

Yes

Reviewer Expertise:

The research results are important as they will form the basis for precautions to be taken for HD patients in possible future pandemics.

I confirm that I have read this submission and believe that I have an appropriate level of expertise to confirm that it is of an acceptable scientific standard.

# Reviewer report: Reviewer response for version 1

**DOI:** 10.5256/f1000research.174077.r346186

**Published:** 2025-02-05

**Authors:** Mehtap Kavurmaci

**Affiliations:** 1 Nursing Faculty, Department of Internal Medicine Nursing, Atatürk University, Erzurum, Turkey

Dear Authors,

 The findings of this research examining the experiences of hemodialysis patients during the COVID-19 pandemic offer applicable recommendations for nursing practice and health policies. The data were collected using the in-depth interview method. The research results show that hemodialysis patients developed adaptive coping strategies during the COVID-19 pandemic, but their need for support from healthcare providers continues.

 The recommendations that I think will contribute to the research are listed below.

 1- It is recommended to provide information about adaptive coping strategies in the introduction section of the research. This information will contribute to the understanding of the findings by the readers.

 2- The fact that the research is limited to only two hospitals may limit the generalizability of the findings. However, it was appropriate that you stated this in the limitations section.

 3- The phone interviews conducted due to COVID-19 may have missed some important observations that could have been obtained in face-to-face interviews. Please state this situation in more detail in the limitations section, including how many people and how many phone interviews were conducted.

 4- There are studies in the literature examining the effects of Covid-19 on Hemodialysis patients. To enrich the discussion section, I recommend that the resources I suggested be read and used in the discussion section and added to the references.

 (Kosunalp N, Kavurmaci M. Determination of anxiety, depression, avoidance, and obsessions experienced by hemodialysis patients during the COVID-19 period. Ther Apher Dial. 2023 [Ref - 1];27(6):1070–8. https://doi.org/10.1111/1744-9987.14031)

 (Nadort E, Rijkers N, Schouten RW, Hoogeveen EK, Bos W, Vleming LJ, et al. Depression, anxiety, and quality of life of hemodialysis patients before and during the COVID-19 pandemic. J Psychosom Res. 2022 [Ref - 2];158:110917. https://doi.org/10.1016/j.jpsychores.2022.110917)

 (Dehghan M, Namjoo Z, Mohammadi Akbarabadi F, Fooladi Z, Zakeri MA. The relationship between anxiety, stress, mental health and mindfulness in patients undergoing hemodialysis: A survey during the COVID-19 outbreak in Southeastern Iran. Health Sci Rep. 2021 [Ref - 3];4(4):e461. https://doi.org/10.1002/ hsr2.461)

Is the work clearly and accurately presented and does it cite the current literature?

Partly

If applicable, is the statistical analysis and its interpretation appropriate?

Yes

Are all the source data underlying the results available to ensure full reproducibility?

Yes

Is the study design appropriate and is the work technically sound?

Partly

Are the conclusions drawn adequately supported by the results?

Yes

Are sufficient details of methods and analysis provided to allow replication by others?

Yes

Reviewer Expertise:

The research results are important as they will form the basis for precautions to be taken for HD patients in possible future pandemics.

I confirm that I have read this submission and believe that I have an appropriate level of expertise to confirm that it is of an acceptable scientific standard, however I have significant reservations, as outlined above.

## References

[1] : Determination of anxiety, depression, avoidance and obsessions experienced by hemodialysis patients during the COVID-19. *Ther Apher Dial* .2023;27(6) : 10.1111/1744-9987.14031 1070-1078 10.1111/1744-9987.14031 37386825

[2] : Depression, anxiety and quality of life of hemodialysis patients before and during the COVID-19 pandemic. *J Psychosom Res* .2022;158: 10.1016/j.jpsychores.2022.110917 110917 10.1016/j.jpsychores.2022.110917 35462121 PMC9008976

[3] : The relationship between anxiety, stress, spiritual health, and mindfulness among patients undergoing hemodialysis: A survey during the COVID-19 outbreak in Southeast Iran. *Health Sci Rep* .2021;4(4) : 10.1002/hsr2.461 e461 10.1002/hsr2.461 34938901 PMC8670730

# Response

**Published:** 2025-09-12

**Authors:** Dewiyanti Toding

**Affiliations:** Dialysis Unit, Hasanuddin University Hospital, Makassar, South Sulawesi, Indonesia

**Response 1:**

 We agree with the reviewer’s suggestion. We have added a description of adaptive coping strategies in the *Introduction (page 3, paragraph 3)* to provide readers with a clearer understanding of the context and relevance of coping mechanisms for hemodialysis patients during the pandemic.

**Response 2:**

 Thank you for this valuable point. We have emphasized this issue in the *Limitations (page 13, paragraph 1)*, highlighting that the recruitment of participants from only two hospitals restricts the generalizability of the findings.

**Response 3:**

 We appreciate this comment. We have revised the *Limitations (page 13, paragraph 1)* to clarify that 11 interviews were conducted by telephone and 4 were conducted face-to-face. We also noted that the use of telephone interviews may have limited the ability to capture nonverbal cues and contextual observations.

**Response 4:**

 We thank the reviewer for these useful references. We have incorporated the suggested studies into the *Discussion*:

- *Page 10, paragraph 2* → Kosunalp & Kavurmaci (2023) and Nadort et al. (2022) were added to strengthen the discussion on the psychological impacts (anxiety, depression) of hemodialysis patients during COVID-19.
- *Page 11, paragraph 2* → Dehghan et al. (2021) was added to support our findings on coping strategies and mindfulness among hemodialysis patients.
- *Page 12, paragraph 1* → Nadort et al. (2022) and Kosunalp & Kavurmaci (2023) were added again to reinforce the role of nurses in addressing psychosocial needs.

These references have also been included in the reference list.

# Reviewer report: Reviewer response for version 1

**DOI:** 10.5256/f1000research.174077.r342952

**Published:** 2025-01-16

**Authors:** Natasha Hubbard Murdoch

**Affiliations:** 1 University of Saskatchewan, Saskatoon, Saskatchewan, Canada

This qualitative descriptive study presents themes on the experience of 15 patients with end-stage renal disease in Indonesia during the COVID-19 pandemic. This nursing research focused on those patients in hemodialysis. The researchers presented a balanced view of the concerns hemodialysis patients face in comparison to the exacerbated concerns because of the pandemic. The themes described the emotional and behavioral responses to the pandemic, the physical, psychological, social and economic impacts, and the adaptive and maladaptive coping mechanisms of having hemodialysis during COVID. The focus of the discussion was on how nurses can ensure holistic provision of care to specifically support coping skills. This research is unique in that it presents findings from a nursing specialty that provides critical insight into the relationship with patients and families experiencing end-stage renal disease.

**Major comments:**

 Please describe qualitative descriptive design more thoroughly and rationalize its use for this particular study.

 “The determination of participants is based on data adequacy” (p. 3).  Clarify this decision, specifically what data adequacy means. A reference is required.

 “Once data saturation is reached, whereby no new insights or information can be derived from the participants” (p. 3). The Bradshaw et al. reference provides a great description of the rationales for data saturation, but the argument for its use was not provided in this article. Considering that participants were recruited from two hospitals using data collection methods differently, explain how saturation can be affected or met.

 There were two instances where the principal researcher noted they gained practical experience with data collection: 1) “She has also practised conducting interviews with hemodialysis patients, though not with the specific participant” (p. 3), and 2)“The outcomes of these preliminary interviews were documented and reviewed with the supervisors” (p. 4). These are great acknowledgements to the school, but unless they refer specifically to data that contributes to this study, these comments should be removed. In acknowledgment of the relationship with the research team members, the authors may choose to note this in a section about rigor.

 “Roy’s theory,” (p. 11). The authors allude to the integration of nursing theory late in the paper without any explanation or reference. At minimum, cite. However, it would be prudent to include a description of Roy’s theory and how the lens is employed during data analysis in the methodology section. This would also add some credence to what is presented in the discussion.

 This sentence in the discussion is a random reference to ‘personality’ that has not been addressed anywhere else in the paper. This must be addressed earlier, or it must be addressed more thoroughly prior to bringing it up in this way. “This is relate to the research conducted by Bacon & Corr (2020) and Carvalho et al. (2020) state that personality functions influence adaptive and coping strategies related to health problems during the COVID-19 pandemic.” (p. 11)

 The references in the discussion could be updated to reflect a more recent reality of COVID. The authors may consider writing a reflective section on how the evidence has changed from the time of this study to what has since been reported for this specialty.

**Minor comments:**

 The paper requires a rigor section which is a required component of qualitative research. Rather than presenting this as a student paper, the following statements should be reworded to reflect rigor criteria: 1) “The research proceeded only after obtaining supervisor approval to continue the interviews.” (p. 4), and 2) “Two experts oversaw the research, and its validity was assessed through a thesis examination involving two expert examiners and nursing master’s students as reviewers. This comprehensive process ensured the research’s quality and reliability.” (p. 4), and 3) “The findings were shared with participants for verification.” (p. 4). Please ensure to follow a rigor framework such as Lincoln and Guba (or others).

 Allusion to a purpose of providing suggestions for nursing interventions but the implications section is superficially written. Articulate specific clinical practice interventions. “Therefore, the researchers aimsto capture these perspectives to develop more suitable nursing interventions for them..” (p. 3)

 Subtheme, physical impact.  End of page 6. Incongruent narrative in relation to the data provided. The pain of swabs is not as significant as the pain of fluid build-up. “The subsequent physical impact is pain. The mandatory (p. 6) and, “swab obligations carried out by participants are a cause of pain, which is necessary for them to continue undergoing the hemodialysis process” (p. 7)

**General writing concerns:**

 The paper requires an edit for English spelling, grammar, and structure. The following are the suggestions I can offer.

 “aims” (p. 1) aimed – the study is done

 Repetitive sentence twice in a row. “As a risk group, hemodialysis patients have a fear of not being able to face COVID-19 if they are exposed. As a high-risk group, hemodialysis patients fear they may not be able to cope with COVID-19 if exposed (WHO, 2020a).” (p. 3)

 Quite a few occurrences where words, sentences, or concepts are repeated multiple times in a sentence or paragraph. For conciseness, please reword.

 : “Hemodialysis patients must go to the unit hemodialysis 2-3 times per week and undergo 4-5 hour hemodialysis process, further increasing the patient’s risk of being exposed to COVID-19” (p. 3)

 : “In addition, some patients’ schedules have been modified as a result of the hemodialysis unit’s efforts to optimize the hemodialysis schedule.” (p. 6)

 : “The majority of participants reported experiencing disturbances in their sleep patterns during the pandemic. However, during the current pandemic, these disturbances have been exacerbated due to concerns about the results of the swab.” (p. 7)

 :“A reduction in social interaction and a sense of ostracism were among the social impacts experienced by participants. A reduction in social interaction has been observed among fellow hemodialysis patients in the hemodialysis unit. Other social impacts include instances of ostracism. Some participants were ostracised by their families and neighbours during the pandemic due to the assumption that they had contracted the COVID-19 virus from the hospital.” (p. 8)

 “also a physical” (p. 6) This sentence requires rewriting or a supporting reference. Is there evidence that BP increases in end-stage renal clients ONLY in relation to anxiety about COVID? Statements such as these need to be well cited.

**Confusing wording or edits :**

 “face patients” (p. 3). Clarify.

 “aimsto” (p. 3) aimed?

 “and proficient” (p. 3) missing a word

 “and telephone” (p. 4) This sentence is unclear.

 “in relation to” (p. 5) because of? Anxiety requires a cause/effect wording

 “arises” (p. 5) arose – past tense

 “It should be noted that swab results were not available.” (p. 6) This sequence of sentences, the one before and after, do not relate to this one. Please describe whether swab results, when not ready on time, affected schedules. This is a set of confusing statements in relation to the sequence of swabbing that seemed to be regularly scheduled at every two to three weeks.

 This translation within a participant quote requires clarification. Not sure a patient would say, expressing dissatisfaction. “expressing dissatisfaction” (p. 8)

 Curious how maladaptive coping mechanisms is described since it has not been defined in the methods nor described in the discussion. “others resorted to maladaptive coping mechanisms to deal with the pandemic’s multifaceted impacts.” (p. 9)

 “include” (p. 9) missing wording

 “Entire” (p. 9) all?

 “which” (p. 9) delete?

 “Hemodialysis schedule were overcrowding” (p. 10) reword this sentence

 “negative behavior” (p. 10) What is negative behaviour? This needs rewording. Risky? Emotional responses are protective so it’s difficult to put a qualifier to how it is expressed without knowing the demographics and context.

 “They done it t” (p. 10). Not grammatically correct

 “Therefore, if participants already have symptoms such as fever, cough, and shortness of breath, participants will chose to treat themselves.” (p. 10). This statement is not supported by evidence, nor is it explained well in the ensuing sentences. What does this mean in relation to hemodialysis treatment and/or COVID?

 “When” (p. 10). This word delineates the start of a new paragraph and new set of commentary on misinformation.

 “Chronic, especially those requiring routine or long-term treatment.” (p. 10). Not a sentence

 “they infected” (p. 10) sentence structure

 “The World Health Organization (WHO)” (p. 10). The acronym was used in a previous paragraph.

 This both does not apply and does not reflect the evidence from the pandemic. This is an example where references need to be updated to acknowledge when the data was collected, but how the paper impacts current reality. “actively trying to control and reduce the impact of this pandemic by identifying, testing, and treating infected patients and developing drugs, vaccines, and treatment protocols (WHO, 2020b).

 Purpose of this statement? “However, success in overcoming the pandemic cannot be confirmed (Kumar & Nayar, 2020).” (p. 11)

 This is a random intervention that is not written within other options or within its own section. Provide an evidentiary citation that this is an option. “Therefore, hemodialysis nurses can create chat groups that are innovative, engaging, and easily accessible to hemodialysis patients and families.” (p. 11)

 Some of the expenses suggested by the authors are minimized in statements such as this. Economic impacts certainly included PPE, swabs, and transportation, but probably also included changes to wages, benefits, ability to access appropriate diets… “mainly by supplying funds for purchasing personal protective equipment. Apart from that, swabs and other examinations are mandatory.” (p. 11)

 The spiritual connection was a surprise under stressors. This phrase in particular is colloquial. “get used to it” (p. 11)

 What is like? “It is like” (p. 11)

 Not sure that parenting should be presented as a burden. “and the burden of being parents” (p. 11)

 Starting with the sentence “Understanding” (p. 11) what is strategy coping?

 “consist” (p. 12) that consist of?

 “underwent” (p. 12) Who underwent?

Is the work clearly and accurately presented and does it cite the current literature?

Partly

If applicable, is the statistical analysis and its interpretation appropriate?

Not applicable

Are all the source data underlying the results available to ensure full reproducibility?

No

Is the study design appropriate and is the work technically sound?

Yes

Are the conclusions drawn adequately supported by the results?

Yes

Are sufficient details of methods and analysis provided to allow replication by others?

Partly

Reviewer Expertise:

Nursing, pandemic experiences for nursing disciplines including educators, practice, administration in licensed practical nursing, psychiatric nursing and registered nursing in addition to the unregulated nursing pandemic experience for continuing care assistants or personal support workers

I confirm that I have read this submission and believe that I have an appropriate level of expertise to confirm that it is of an acceptable scientific standard, however I have significant reservations, as outlined above.

# Response

**Published:** 2025-02-02

**Authors:** Dewiyanti Toding

**Affiliations:** Dialysis Unit, Hasanuddin University Hospital, Makassar, South Sulawesi, Indonesia

*Dear Editors and Reviewers,*

*We thank for your thorough reading of our manuscript, comments and suggestions that helped us to improve the manuscript. As indicated below, we have tried to do our best to respond to all the queries. All the revisions & answers are stated below:*

***Major Comments:***

 Comment 1 : Please describe qualitative descriptive design more thoroughly and rationalize its use for this particular study.

 Answer 1 : A description about qualitative descriptive design was added to the methods and study design.

 Comment 2 : “The determination of participants is based on data adequacy” (p. 3).  Clarify this decision, specifically what data adequacy means. A reference is required.

 Answer 2 : An explanation about data adequacy was added in the paragraph, in the method section, setting and participants. With the statement below:

*“Adequate data refers to data that is sufficient to address the research questions, indicated by the achievement of data saturation, where no new information or insights can be obtained from the participants”*

*Comment 3 : *“Once data saturation is reached, whereby no new insights or information can be derived from the participants” (p. 3). The Bradshaw et al. reference provides a great description of the rationales for data saturation, but the argument for its use was not provided in this article. Considering that participants were recruited from two hospitals using data collection methods differently, explain how saturation can be affected or met.

*Answer 3 : *Participants were recruited from two hospitals using the same data collection method, explanation about how saturation can be affected or met, was added in method section, setting and participants, below :

 “ *Data saturation was determined independently at each location, based on the absence of new information that aligns with the unique characteristics of participants*”

 Comment 4 : There were two instances where the principal researcher noted they gained practical experience with data collection: 1) “She has also practised conducting interviews with hemodialysis patients, though not with the specific participant” (p. 3), and 2)“The outcomes of these preliminary interviews were documented and reviewed with the supervisors” (p. 4).

 These are great acknowledgements to the school, but unless they refer specifically to data that contributes to this study, these comments should be removed. In acknowledgment of the relationship with the research team members, the authors may choose to note this in a section about rigor.

 Answer 4 : The comments about how the author gained practical experience was already been removed, and added to rigor’s explanation, at section of method, data collections, stated below:

 “ *Rigor of this research was using framework of Lincoln and Guba (* *Gunbayi, 2024* *). Credibility implemented by conducted preliminary interviews with three patients who were not part of the study. Researcher spent 6 weeks in average in each site to gain thrustworthies with all participants.* *The interviewer was the first author, a dialysis nurse with 10 years of experience. She holds certification in dialysis nursing competency, master of nursing degree, has completed qualitative research courses and proficient in data collection through interviews*. *Two experts oversaw the research, and its validity was assessed through a thesis examination conducted by two expert examiners,with nursing master's students serving as reviewers. This comprehensive process ensured the research's quality and reliability.* *The research proceeded only after obtaining expertise approval to continue the interviews.* *Transcribed data were returned to participants for confirmation and clarification before being processed further.* *To ensure transferability in this research, we provided clear and detailed information about the content about the study. Additionally, all statements originally made in local languages* *have been translated into English to ensure that the research is accessible and understandable to all readers.”*

*Comment 5 : *“Roy’s theory,” (p. 11). The authors allude to the integration of nursing theory late in the paper without any explanation or reference. At minimum, cite. However, it would be prudent to include a description of Roy’s theory and how the lens is employed during data analysis in the methodology section. This would also add some credence to what is presented in the discussion.

*Answer 5 : S*ource of citations was added, in page 11 and also in the references:

 Roy, C.(2009). The Roy Adaptation Model. 3. Upper Saddle River, NJ: Pearson Education, Inc.

 And explanation about Roy’s adaptation theory is already stated in part of discussion.

*Comment 6 : *This sentence in the discussion is a random reference to ‘personality’ that has not been addressed anywhere else in the paper. This must be addressed earlier, or it must be addressed more thoroughly prior to bringing it up in this way. “This is relate to the research conducted by Bacon & Corr (2020) and Carvalho et al. (2020) state that personality functions influence adaptive and coping strategies related to health problems during the COVID-19 pandemic.” (p. 11)

*Answer 6 : *The differences in participants' personalities could be a factor that influences coping strategies in this research. But eventually we decided that it wasn't the point of research’s discussion and so it didn’t fully explained, but we decided to keep it, rather as a supporting opinions regarding the factors that influence coping strategies.

*Comment 7 : *The references in the discussion could be updated to reflect a more recent reality of COVID. The authors may consider writing a reflective section on how the evidence has changed from the time of this study to what has since been reported for this specialty.

*Answer 7 : *Explanation about current condition and previous outbreak, was added in the limitation section “ *During the period when this study was conducted, compared with the current context, the COVID-19 pandemic as the issue of this research, has changed significantly. Because it’s no longer as severe as it was. However, the findings from this research provide valuable new insights into the needs of hemodialysis patients when dealing with challenging condition*”

***Minor Comments:***

*Comment 8 : *The paper requires a rigor section which is a required component of qualitative research. Rather than presenting this as a student paper, the following statements should be reworded to reflect rigor criteria: 1) “The research proceeded only after obtaining supervisor approval to continue the interviews.” (p. 4), and 2) “Two experts oversaw the research, and its validity was assessed through a thesis examination involving two expert examiners and nursing master’s students as reviewers. This comprehensive process ensured the research’s quality and reliability.” (p. 4), and 3) “The findings were shared with participants for verification.” (p. 4). Please ensure to follow a rigor framework such as Lincoln and Guba (or others).

*Answer 8 : *An explanation about rigor was added in the paragraph, following Lincoln and Guba framework (credibility, transferability, dependability and confirmability) in the section of method, data collections.

*Comment 9 : *Allusion to a purpose of providing suggestions for nursing interventions but the implications section is superficially written. Articulate specific clinical practice interventions. “Therefore, the researchers aimsto capture these perspectives to develop more suitable nursing interventions for them..” (p. 3)

*Answer 9 : *Nursing implication was explained in the discussion section, in the 8 ^th^ – 11 ^th^ paragraph

*Comment 10 : *Subtheme, physical impact.  End of page 6. Incongruent narrative in relation to the data provided. The pain of swabs is not as significant as the pain of fluid build-up. “The subsequent physical impact is pain. The mandatory (p. 6) and, “swab obligations carried out by participants are a cause of pain, which is necessary for them to continue undergoing the hemodialysis process” (p. 7)

*Answer 10 : *Explanations have been added to clarify what participants stated (P6 and P7).

**General writing concerns:**

*Comment 11 : *The paper requires an edit for English spelling, grammar, and structure. The following are the suggestions I can offer.

*Answer 11 : *Thank you so much for your kind suggestion & guidance, we've tried to revise the manuscript according to your suggestions. Hope the quality of spelling, grammar, and structure will be much improved and hope you agree

*Comment 12: *“aims” (p. 1) aimed – the study is done

 Repetitive sentence twice in a row. “As a risk group, hemodialysis patients have a fear of not being able to face COVID-19 if they are exposed. As a high-risk group, hemodialysis patients fear they may not be able to cope with COVID-19 if exposed (WHO, 2020a).” (p. 3)

*Answer 12 : *Those repetitive sentences have been deleted

*Comment 13 : *Quite a few occurrences where words, sentences, or concepts are repeated multiple times in a sentence or paragraph. For conciseness, please reword.

 : “Hemodialysis patients must go to the unit hemodialysis 2-3 times per week and undergo 4-5 hour hemodialysis process, further increasing the patient’s risk of being exposed to COVID-19” (p. 3)

*Answer 13 : *Repetitive words, sentences or concepts have been removed and adjusted.

*Comment 14 : *“In addition, some patients’ schedules have been modified as a result of the hemodialysis unit’s efforts to optimize the hemodialysis schedule.” (p. 6)

*Answer 14 : *Repetitive words have been removed and adjusted.

*Comment 15 : *“The majority of participants reported experiencing disturbances in their sleep patterns during the pandemic. However, during the current pandemic, these disturbances have been exacerbated due to concerns about the results of the swab.” (p. 7)

*Answer 15 : *Repetitive words have been removed and adjusted.

*Comment 16 : *“A reduction in social interaction and a sense of ostracism were among the social impacts experienced by participants. A reduction in social interaction has been observed among fellow hemodialysis patients in the hemodialysis unit. Other social impacts include instances of ostracism. Some participants were ostracised by their families and neighbours during the pandemic due to the assumption that they had contracted the COVID-19 virus from the hospital.” (p. 8)

*Answer 16 : *Repetitive sentences have been removed and adjusted.­­­­­

*Comment 17 : *“also a physical” (p. 6) This sentence requires rewriting or a supporting reference. Is there evidence that BP increases in end-stage renal clients ONLY in relation to anxiety about COVID? Statements such as these need to be well-cited

*Answer 17 : *The sentence adjusted into “ *An **increase in blood pressure was found as a physical impact** experienced by hemodialysis patients during COVID-19 pandemic due to anxiety about swab results*” referred to participant's statement (P2), below :

 “ *The **swab test is what scares me, and it causes my blood pressure to rise**. It is always on my mind, especially when waiting for the results—my results are coming out tomorrow, and I am so anxious"(P2)” and already stated in page 7.*

**Confusing wording or edits :**

 We apologize for the confusion. All the revisions are stated below:

*Comment 18 : *“face patients” (p. 3). Clarify.

*Answer 18 : *This sentence “ *Transportation to the hospital is also a challenge, as many hemodialysis patients have to face patients who depend on public transportation*” have already changed into “ *Transportation to the hospital is also a challenge, as many hemodialysis patients have to face the reality that they depend on public transportation*”

*Comment 19 : *“aimsto” (p. 3) aimed?

*Answer 19 : *The word “ *Aimsto*” was replaced with “ *Aimed*”

*Comment 20 : *“and proficient” (p. 3) missing a word

*Answer 20 : *This sentence in page 3 was deleted due to its part of rigour, and rewritten in the discussion as part of rigour.

*Comment 21 : *“and telephone” (p. 4) This sentence is unclear.

*Answer 21 : *This sentence “ *Researchers conducted face-to-face interviews three times **and telephone** due to the COVID-19 pandemic situation*” have already change into “ *Researchers conducted three times contact, **either face-to-face interviews or telephone calls** due to the COVID-19 pandemic situation*”

*Comment 22 : *“in relation to” (p. 5) because of? Anxiety requires a cause/effect wording

*Answer 22 : *This sentence “Some participants indicated that they experienced feelings of anxiety **in relation to both fellow hemodialysis patients and hemodialysis nurses**” have already change into “Some participants indicated that they experienced feelings of anxiety **because of being suspected by other patients or the nurses**, that they may be carrying the SARS-CoV-2 virus”

*Comment 23 : *“arises” (p. 5) arose – past tense

*Answer 23 : *The word “ *Arise*” was replaced into “ *Arose*”

*Comment 24 : *“It should be noted that swab results were not available.” (p. 6) This sequence of sentences, the one before and after, do not relate to this one. Please describe whether swab results, when not ready on time, affected schedules. This is a set of confusing statements in relation to the sequence of swabbing that seemed to be regularly scheduled at every two to three weeks.

 This translation within a participant quote requires clarification. Not sure a patient would say, expressing dissatisfaction. “expressing dissatisfaction” (p. 8)

*Answer 24 : *This sentence “ *As a consequence of the pandemic, the patient's hemodialysis schedule was postponed. **It should be noted that swab results were not available***” has already been adjusted and given the reason, so it was changed into “ *As a consequence of the pandemic, **the patient's hemodialysis schedules was postponed if*** ***their swab results were not available. In some cases, it was found that the results of the swab were received 1-2 days after swabbing process, due to limitations in resources***”

*Comment 25 : *Curious how maladaptive coping mechanisms is described since it has not been defined in the methods nor described in the discussion. “others resorted to maladaptive coping mechanisms to deal with the pandemic’s multifaceted impacts.” (p. 9)

*Answer 25 : *The word “multifaceted impacts” have been deleted.

 Genuinely, maladaptive coping mechanisms are described by the participant (P3 and P6) by treating themselves without having a consultation with the doctor or using a mask while sleeping even he/she didn’t suffer from COVID-19.

*Comment 26 : *“include” (p. 9) missing wording

*Answer 26 : *The sentence “ *Stress during an infectious disease outbreak **includes** fear and worry about health problems and worsening chronic health problems (CDC, 2020)*” was changed into “ *Stress during an infectious disease outbreak **such as** fear and concern about health problems and the worsening of chronic conditions, can significantly impact individuals (CDC, 2020)*”

*Comment 27 : *“Entire” (p. 9) all?

*Answer 27 : *The word “ *Entire*” was replaced with “ *All participants*”

*Comment 28 : *“which” (p. 9) delete?

*Answer 28 : *The word "which" on page 9 had already been deleted

*Comment 29 : *“Hemodialysis schedule were overcrowding” (p. 10) reword this sentence

*Answer 29 : *This sentence “ ***Hemodialysis schedule were overcrowding*** *and limited health personnel in hemodialysis units which made this situation more challenging to prepare many special machines for hemodialysis patients with COVID-19 at the start of this pandemic*” was changed into “ ***The hemodialysis schedule was often full*** *, and the limited number of healthcare personnel made it challenging to prepare the necessary machines for COVID-19-positive hemodialysis patients*”

*Comment 30 : *“negative behavior” (p. 10) What is negative behaviour? This needs rewording. Risky? Emotional responses are protective so it’s difficult to put a qualifier to how it is expressed without knowing the demographics and context.

*Answer 30 : *Negative behaviour was explained in the added sentence " *Excessive emotional responses can lead to negative behaviours. COVID-19, as a new infectious disease, has triggered heightened anxiety and fear, which can result in dangerous behaviours (Ho et al (2020)”*

*Comment 31 : *“They done it t” (p. 10). Not grammatically correct

*Answer 31 : *This sentence “ *Participants chose to* *hide their health status, mainly if they got symptoms that were similar to the main symptoms of COVID-19 or had a history of contact with families who have COVID-19 symptoms. **They did it** to avoid referrals to an isolation room and carrying out COVID-19 screening which could later have an impact on delay…*” changed into *“Participants chose to hide their health status, particularly when they exhibited symptoms similar to those of COVID-19 or had a history of contact with infected family members. **This was done to avoid** being referred to isolation rooms or undergoing COVID-19 screenings, which could cause delays in their hemodialysis schedules”*

*Comment 32 : *“Therefore, if participants already have symptoms such as fever, cough, and shortness of breath, participants will chose to treat themselves.” (p. 10). This statement is not supported by evidence, nor is it explained well in the ensuing sentences. What does this mean in relation to hemodialysis treatment and/or COVID?

*Answer 32 : *The sentences adjusted into “ *As a result, participants with symptoms like fever, cough, and shortness of breath chose to self-medicate*” was a statement spoken by participants number 3 (P3).

 This condition is stated on page 9 “ *When I got fever, cough or shortness of breath, I took vitamin C,  folic acid and played sports despite the rain. I also drank honey and boiled ginger, which is high in potassium, but I do not care. The main thing is that it is clear: do not be exposed to COVID-19” (P3)”*

*Comment 33 : *“When” (p. 10). This word delineates the start of a new paragraph and new set of commentary on misinformation.

*Answer 33 : *This word " ***when*** " was changed into *" **At the onset of a crisis or pandemic**, individuals and communities typically seek information about the situation. However, when official sources or trusted news are lacking, people often turn to social media or other unreliable sources, which can fuel negative emotional responses and amplify fear, uncertainty, and distorted risk perceptions (Torales et al., 2020)”*

*Comment 34 : *“Chronic, especially those requiring routine or long-term treatment.” (p. 10). Not a sentence

*Answer 34 : *The sentence " ***Chronic,*** *especially those requiring routine or long-term treatment*" paraphrased to *"* ***An especially ironic situation arose*** *for hemodialysis patients, who require routine or long-term treatment. This study found that the COVID-19 pandemic negatively impacted the physical health of kidney failure patients undergoing hemodialysis, primarily due to delays and changes in their treatment schedules. In addition to the physical health impacts, the pandemic has also had significant mental health consequences (Pfefferbaum & North, 2020)”*

*Comment 35 : *“they infected” (p. 10) sentence structure

*Answer 35 : the s*entence " *they infected*" replaced with " *exposed to*"

*Comment 36 : *“The World Health Organization (WHO)” (p. 10). The acronym was used in a previous paragraph.

*Answer 36 : *The abbreviation of WHO is deleted to avoid repetition

*Comment 37 : *This both does not apply and does not reflect the evidence from the pandemic. This is an example where references need to be updated to acknowledge when the data was collected, but how the paper impacts current reality. “actively trying to control and reduce the impact of this pandemic by identifying, testing, and treating infected patients and developing drugs, vaccines, and treatment protocols (WHO, 2020b).

*Answer 37 : *The sentence was changed into *“Currently, By the time of initial outbreak, the World Health Organization is actively trying to control and reduce the impact of this pandemic by identifying, testing, and treating infected patients and developing drugs, vaccines, and treatment protocols. However, success in overcoming this pandemic could not be guaranteed at that time due to the unpredictability of the situation (Kumar & Nayar, 2020)”.*

 This was to highlight that the action happened at the start of the pandemic situation, not currently as stated before.

*Comment 38 : *Purpose of this statement? “However, success in overcoming the pandemic cannot be confirmed (Kumar & Nayar, 2020).” (p. 11)

*Answer 38 : *The sentence already rewrote into *"However, success in overcoming this pandemic could not be guaranteed at that time due to the unpredictability of the situation (Kumar & Nayar, 2020)”*

*Comment 39 : *This is a random intervention that is not written within other options or within its own section. Provide an evidentiary citation that this is an option. “Therefore, hemodialysis nurses can create chat groups that are innovative, engaging, and easily accessible to hemodialysis patients and families.” (p. 11)

*Answer 39 : *The sentence rewrote into *“Therefore, hemodialysis nurses could consider creating innovative, engaging, and easily accessible chat groups for hemodialysis patients and their families”*

 The purpose of creating chat group by nurses for hemodialysis patients during the pandemic was intended to enabled hemodialysis patients to continue communicating, share stories or provide support to each other through the group chat. Apart from that, families of hemodialysis patients can also share stories and support each other on how to deal with and care for hemodialysis patients during the COVID-19 pandemic.

*Comment 40 : *Some of the expenses suggested by the authors are minimized in statements such as this. Economic impacts certainly included PPE, swabs, and transportation, but probably also included changes to wages, benefits, ability to access appropriate diets… “mainly by supplying funds for purchasing personal protective equipment. Apart from that, swabs and other examinations are mandatory.” (p. 11)

*Answer 40 : *The additional costs that had to be borne were mainly personal protective equipment, because hospitals had to implement protection protocols during hemodialysis services. Meanwhile, wages, benefits, and the ability to access appropriate diets could have been an additional burden for patients, but were not a concern for researchers.

*Comment 41 : *The spiritual connection was a surprise under stressors. This phrase in particular is colloquial. “get used to it” (p. 11)

*Answer 41 : *Paraphrased this sentence into " *During the pandemic, the majority of participants employed adaptive coping strategies such as spiritual strengthening, positive thinking, and efforts to accept and acclimatize to their circumstances.*"

*Comment 42 : *What is like? “It is like” (p. 11)

*Answer 42 : *Word "it's like" was replaced with "Some of the examples were"

*Comment 43 : *Not sure that parenting should be presented as a burden. “and the burden of being parents” (p. 11)

*Answer 43 : *“Several previous studies identified factors of demographics as a potential risk of implementing maladaptive coping strategies in dealing with the COVID-19 pandemic, such as age, gender, race, socioeconomic status, and the burden of being parents.”

 This sentence is quoted from Atchison et al (2020) and also Clay & Parker (2020) and is not the opinion of the researcher.

*Comment 44 : *Starting with the sentence “Understanding” (p. 11) what is strategy coping?

*Answer 44 : *This sentence "Understanding of strategy coping" needs more explanation, so we changed into " *Efforts by trying to understand strategies in managing coping mechanisms is very important to support the patient's coping efforts "*

*Comment 45 : *“consist” (p. 12) that consist of?

*Answer 45 : *This sentence “ *Results of research on end-stage renal disease patients undergoing hemodialysis in the era of the COVID-19 pandemic illustrate three main themes, consist of…*” already changed into " *Results of research on end-stage renal disease patients undergoing hemodialysis in the era of the COVID-19 pandemic illustrate three main themes, namely..*"

*Comment 46 : *“underwent” (p. 12) Who underwent?

*Answer 46 : *This sentences “ *These findings showed that end-stage renal disease patients underwent hemodialysis in the COVID-19 pandemic era….*” is clearly written into " *These findings demonstrated in patients with end-stage renal disease who underwent hemodialysis during the pandemic era*"

 We have conveyed all our answers above, and hope you can provide further feedback, we thank you for your attention and look forward to receiving your further communications.

 Yours sincerely,

 Dewiyanti Toding
